# Cryptotanshinone inhibits *Staphylococcus aureus* NdhC via uncompetitive inhibition: Collateral sensitivity in respiratory-deficient SCVs

**DOI:** 10.1080/21505594.2026.2723683

**Published:** 2026-08-24

**Authors:** Zheng-Jie Xue, Ming-Yu Huang, Jun-Feng Wang, Si-Yi Zhong, Zheng-Cheng Yang, Jian Xu, Bo Jin, Ni-Pi Chen, Chao-Dong Qian

**Affiliations:** College of Life Sciences, Zhejiang Chinese Medical University, Hangzhou, China

**Keywords:** *Staphylococcus aureus*, small colony variants (SCVs), cryptotanshinone (CT), NdhC, NDH-2, antimicrobial resistance, collateral sensitivity, uncompetitive inhibition

## Abstract

*Staphylococcus aureus* small colony variants (SCVs) pose a clinical challenge due to antibiotic tolerance and recurrent infections. Targeting the bacterial respiratory chain offers a potential strategy to overcome SCV-mediated resistance. Here we show that cryptotanshinone (CT) selectively inhibits *S. aureus*’s NDH-2 isoform NdhC. Antimicrobial assays showed that *ndhC* overexpression doubled bacterial sensitivity to CT, while *ndhF* overexpression had no effect. *In vitro* enzymatic analysis indicated that CT inhibits NdhC, with noncompetitive inhibition against NADH and uncompetitive inhibition against menadione (MD). Molecular docking and site-directed mutagenesis identified threonine T352 as critical for CT binding to the NdhC-MD complex. Using membrane vesicle assays and cellular thermal shift assays, we obtained evidence that CT acts at NdhC in isolated membrane preparations in a substrate-dependent manner. Notably, gentamicin-induced SCVs carrying *fusA* and *perR* mutations exhibited collateral sensitivity to NDH-2 inhibitors. Further characterization of menaquinone (MK) auxotrophic SCVs, which harbor a frameshift mutation in *menC* and show reduced but detectable NADH dehydrogenase activity, revealed that these variants also display heightened susceptibility to CT, consistent with the notion that residual NdhC activity in respiratory-compromised cells remains targetable. Together, these findings provide insights into the molecular targeting of NdhC by CT and support the concept that the metabolic vulnerability of SCVs may be exploitable for the treatment of refractory *S. aureus* infections.

## Introduction

*Staphylococcus aureus* is a clinically significant Gram-positive pathogenic bacterium that causes a wide spectrum of infections, ranging from superficial skin and soft tissue infections to life-threatening systemic illnesses. The therapeutic efficacy of conventional antibiotics has been progressively compromised by the global dissemination of multidrug-resistant strains, most notably methicillin-resistant *S. aureus* (MRSA). Of greater clinical concern, *S. aureus* readily gives rise to small colony variants (SCVs) upon exposure antibiotic pressure or within the host immune microenvironment. Characterized by retarded growth and attenuated metabolic activity, these variants exhibit a well-documented ability to persist within host cells, and have been associated with the development of chronic and recurrent staphylococcal infections [1,2]. Given that the World Health Organization has classified *S. aureus* as a high-priority pathogen [3], there is an urgent need to develop novel antimicrobial agents with distinct mechanisms of action that can efficiently eradicate SCVs.

Substantial progress has been achieved with strategies targeting the bacterial respiratory chain in anti-tuberculosis therapy. The clinical feasibility of this pathway has been demonstrated by the successful launch of bedaquiline, the first ATP synthase inhibitor [4–7]. However, in contrast to *Mycobacterium tuberculosis*, drug development targeting the respiratory chain components of *S. aureus* remains at a relatively early exploratory stage. Despite slow progress, this pathway has been regarded as a highly promising candidate for development, due to the unique physiological characteristic of *S. aureus* that lacks Complex I and relies primarily on type II NADH dehydrogenases (NDH-2) [8,9]. Notably, emerging evidence indicates that bacterial variants with defective respiratory chain activity, while often resistant to aminoglycoside antibiotics, can exhibit collateral sensitivity to compounds that target energy synthesis, an evolutionary trade-off in which resistance to one antibiotic confers increased susceptibility to other drugs [10–12]. For instance, heme or menaquinone (MK) auxotrophic SCVs display heightened susceptibility to lugdunin [13] and tomatidine [14,15] which act as a cation ionophore and inhibitor of ATP synthase respectively. This energy threshold vulnerability, whereby metabolically compromised variants are less tolerant of additional respiratory chain disruption, may represent a generalizable strategy for targeting drug-tolerant persisters.

Cryptotanshinone (CT), a naturally occurring diterpenoid quinone predominantly distributed in plants of the genus *Salvia*, most abundantly in *Salvia miltiorrhiza*, has been previously characterized as a bacterial respiratory chain inhibitor [16]. Preliminary evidence has suggested that NDH-2 serves as a putative target of this natural compound. Nevertheless, several key scientific questions remain to be addressed, including whether NDH-2 is indeed targeted by CT, and whether this compound can elicit collateral sensitivity effects by exploiting the intrinsic respiratory chain deficiencies of *S. aureus* SCVs. In this study, we first examined whether CT specifically inhibits *S. aureus* NDH-2 through susceptibility assays with *ndh-2*-overexpressing strains and *in vitro* enzymatic analyses. Molecular docking and site-directed mutagenesis further identified key binding residues and provided insights into the kinetic mechanism of inhibition. Using membrane vesicle assays and cellular thermal shift assay (CETSA), we obtained evidence that CT acts at NdhC in isolated membrane preparations in a substrate-dependent manner. Finally, employing a gentamicin-induced and MK-auxotrophic SCV models, we evaluated the antibacterial efficacy of CT against these persistent subpopulation, with further characterization of the MK auxotrophs to assess whether residual NdhC activity is retained under conditions of MK deficiency. Collectively, this study aims to explore whether targeting NDH-2 represents a viable strategy to trigger collateral sensitivity in SCVs with impaired respiratory chain function.

## Materials and methods

### Bacterial strains, plasmids, and chemical reagents

The bacterial strains and plasmids used in this study were described in Table S1. Luria-Bertani (LB) medium (10.0 g/L NaCl (Cat. No. A610476-0001, Sangon Biotech, China), 5.0 g/L yeast extract (Cat. No. P0021B, Oxoid, UK), 10.0 g/L tryptone (Cat. No. LP0042B, Oxoid, UK); pH 7.0) and Tryptic Soy Broth (TSB) medium (Cat. No. LA0020, Solarbio, China) were employed for routine bacterial cultivation, whereas Mueller-Hinton (MH) medium (Cat. No. CM0405B, Oxoid, UK) was used for antimicrobial susceptibility testing. All cultures were incubated at 37°C for 18–24 hours, unless otherwise specified. For plasmid selection and maintenance, the final concentrations of antibiotics used were as follows: 100 μg/mL ampicillin (Cat. No. A800429, Macklin, China), 50 μg/mL kanamycin (Cat. No. K6115, Macklin, China), and 10 μg/mL chloramphenicol (Cat. No. C8050, Solarbio, China). CT (>98% HPLC purity) was obtained from Aladdin (Cat. No. C101974, China), while menadione (MD, >98% HPLC purity, Cat. No. M813096), menaquinone-4 (MK4, >98% HPLC purity, Cat. No. V934192) and reduced nicotinamide adenine dinucleotide (NADH, >98% HPLC purity, Cat. No. N814671) were sourced from Macklin (China). CT was dissolved in dimethyl sulfoxide (DMSO, Cat. No. V87300, Yuanye Bio, China) and subsequently diluted to ensure a final DMSO concentration of <3.2% (v/v).

### Drug susceptibility test

Bacterial susceptibility to antimicrobial agents was assessed by determining the minimum inhibitory concentration (MIC) using the broth microdilution method, following the CLSI guidelines (2018) [17]. Serial two-fold dilutions of antimicrobial agents were prepared in 96-well microplates containing MH medium, followed by the addition of an equal volume of a standardized bacterial suspension at approximately 1.0 × 10^6^ CFU/mL. The microplates were incubated at 37°C under aerobic conditions for 18–20 hours, and the MIC was defined as the lowest concentration of an antimicrobial agent that prevented the visible growth of a microorganism under tested conditions.

### Plasmid construction

To achieve overexpression of *ndhC* and *ndhF*, the *ndhC* and *ndhF* genes were amplified from the chromosomal DNA of *S. aureus* ATCC 25,923 using the primer pairs *ndhC*-F/R and *ndhF*-F/R, respectively. The amplicons were inserted into the plasmid pLI50 (amplified by primers pLI50-F/R) using a one-step seamless cloning kit (Cat. No. AG11808, Accurate Biology, China). Following validation by PCR and DNA sequencing, the recombinant plasmids pLI50-*ndhC*/*ndhF* were transformed into *Escherichia coli* DH5α to generate the strains DH5α-pLI50-*ndhC*/*ndhF*. Plasmids were extracted and subsequently introduced into *S. aureus* RN4220 competent cells by electroporation. After recovery, cells were plated on LB agar containing chloramphenicol, and positive transformants were verified by PCR and DNA sequencing to obtain the overexpression strains RN4220-pLI50-*ndhC/ndhF*. The empty plasmid pLI50 was used to generate the control strain RN4220-pLI50. Primer sequences are listed in Table S2.

Heterologous expression strains were constructed as described above (ref. [18]). Briefly, the *ndhC* and *ndhF* genes were amplified from *S. aureus* 25,923 genomic DNA using the primer pairs *ndhC*-YYBD-F/R and *ndhF*-YYBD-F/R (Table S2), respectively. The resulting fragments were then cloned into the pET28a (+) vector, which was amplified using the primer pair Pet-XXH-F/R, as described above. A 6 × His-tag was fused to the N-terminus of NDH-2 for protein purification. The resulting recombinant plasmids pET28a (+)-*ndhC* and pET28a (+)-*ndhF* were verified by PCR using primers Pet-YZ-F/R (Table S2) and DNA sequencing in *E. coli* DH5α, then transformed into *E. coli* BL21 (DE3).

Site-directed mutagenesis of the *ndhC* gene was performed by whole-plasmid PCR using pET28a (+)-*ndhC* as the template. The primer pairs *ndhC* A319S-F/R, *ndhC* A319E-F/R, *ndhC* T352A-F/R, *ndhC* T352E-F/R, and *ndhC* R385A-F/R (Table S2) were used to amplify the *ndhC*-A319S, *ndhC*-A319E, *ndhC*-T352A, *ndhC*-T352E, and *ndhC*-R385A mutant fragments, respectively. The amplified PCR products were individually assembled using a seamless cloning method, transformed into *E. coli* DH5α, and verified by DNA sequencing using the primer pair *ndhC*-YZ-F/R (Table S2). The correctly sequenced mutant plasmids were subsequently transformed into *E. coli* BL21 (DE3) for recombinant expression.

### Gene knockout

To generate an *ndhF* deletion strain in *S. aureus* Newman, a CRISPR/Cas9-mediated genome editing strategy based on the pnCasSA-BEC plasmid was employed. An sgRNA targeting the *ndhF* locus was designed, and the corresponding complementary oligonucleotides were annealed and ligated into the BsaI-digested pnCasSA-BEC vector using T4 DNA ligase (Cat. No. 2011A, TaKaRa, Japan). Positive clones were screened by PCR using the primer pair sgRNA-F/pnCasSA-R (Table S2) and further confirmed by DNA sequencing. The verified pnCasSA-BEC-sgRNA plasmid was introduced into *S. aureus* RN4220 by electroporation and subsequently transferred into the Newman strain. Transformants were selected on TSA plates supplemented with chloramphenicol (10 μg/mL). Candidate mutants were screened by colony PCR using the primer pair QCYZ-NdhF-F/R (Table S2), and deletion of *ndhF* was further confirmed by DNA sequencing.

### Protein expression and purification

Protein expression and purification were performed as described in reference [18]. Overnight cultures of the heterologous expression strains were diluted and inoculated into LB medium supplemented with kanamycin, followed by shaking at 37°C. Bacterial cells were harvested by centrifugation after cultivation. Cell pellets were resuspended in LB medium containing kanamycin, and isopropyl β-D-1-thiogalactopyranoside (IPTG, 1 M, Cat. No. BS119, Biosharp, China) was added to induce protein expression. Cells were harvested by centrifugation after induction. All purification steps were performed at 4°C. Cell pellets were resuspended in lysis buffer and lysed, followed by sonication and centrifugation to remove insoluble cell debris. Protein samples were loaded onto Ni-NTA columns and incubated overnight at 4°C to allow binding. The columns were then washed and eluted at a natural flow rate to obtain purified protein.

### Enzyme activity assay

Enzyme activity assays were performed as previously described (ref. [18]). The 200 μL reaction mixture contained 50 mM Tris-HCl (pH 7.5; Tris, Cat. No. T8060, Solarbio, China), 300 μM NADH, 20 μM FAD (Cat. No. HY-B1654, MCE, USA), 500 mM NaCl, 5% glycerol (Cat. No. G116203, Aladdin, China), and approximately 0.5 μg (11.34 pmol) of purified NDH-2. Reactions were initiated by adding 30 μM MD. Kinetic constants were determined by supplementing the reaction mixture with 0–400 μM NADH or 0–60 μM MD. The half-maximal inhibitory concentration (IC_50_) values and inhibition kinetics of NDH-2 were determined by incubating the reaction mixture with serial concentrations of the test compound, with 3.2% DMSO as the negative control. NADH dehydrogenase activity in inverted membrane vesicles was determined using the same reaction conditions described above. In this assay, 400 μM NADH was used as the substrate, and inverted membrane vesicles were used as the enzyme source. MD and FAD were not included in the reaction mixture.

### Quantitative polymerase chain reaction

Transcriptional levels of *ndhC* and *ndhF* were analyzed using reverse transcription followed by real-time quantitative PCR (RT-qPCR). A 2 mL bacterial culture was grown at 37°C with shaking (200 rpm) for 16 hours. Cells were harvested by centrifugation at 10,000 × g for 10 minutes, and total RNA was extracted using the SteadyPure RNA Extraction Kit (Cat. No. AG21024, Accurate Biology, China). Complementary DNA (cDNA) was synthesized using the Evo M-MLV RT Mix Kit with gDNA Clean for qPCR (Cat. No. AG11728, Accurate Biology, China). The RT-qPCR was performed using 2 × SYBR Green Abstart PCR Mix (Cat. No. B110031, Sangon Biotech, China) on a qTOWER3 G real-time PCR system (Analytik Jena AG, Germany). Primers used for RT-qPCR are listed in Table S2. Relative transcription levels were calculated using the 2^−ΔΔCT^ method, and all reactions were performed in triplicate.

### Molecular docking simulation

Molecular docking using AutoDock Vina was employed to explore the molecular basis of the interaction between CT and NDH-2. The 3D structure of the small molecule (PubChem CID: 160,254) was obtained from PubChem database (https://pubchem.ncbi.nlm.nih.gov/), and the crystal structure of NDH-2 (PDB ID: 5NA1) from *S. aureus* was obtained from the PDB database (https://www.rcsb.org/).

### Preparation of inverted membrane vesicles

Inverted membrane vesicles were prepared from overnight cultures of *S. aureus*. Bacterial cells were resuspended in KPN buffer (20 mM potassium phosphate, 140 mM NaCl, pH 7.2) and lysed using a French press at 16,000 psi. After low-speed centrifugation at 6,000 rpm for 30 minutes to remove cell debris, the supernatant was ultracentrifuged at 150,000 × g for 40 minutes on an Optima XPN-100 ultracentrifuge (Beckman, USA). The resulting pellets were resuspended and centrifuged at 12,000 rpm for 30 minutes to remove residual contaminants. The supernatant containing purified inverted membrane vesicles was stored at −80°C. The protein concentration of vesicle samples was determined via a BCA protein assay kit (Cat. No. P0012, Beyotime, China) with bovine serum albumin as the standard.

### ATP synthesis assay

The effect of CT on ATP synthesis by inverted membrane vesicles isolated from the indicated *S. aureus* strains was determined using the BacTiter-Glo ATP Detection Kit (Cat. No. C0050S, Beyotime, China). Briefly, the membrane vesicle suspension was diluted to 50 μg/mL in 0.01 M PBS supplemented with 10 mM MgCl_2_, then preincubated with CT for 10 minutes at room temperature. Different energy substrates (NADH, 2.5 mM; succinic acid (Cat. No. 110–15-6, Yuanye Bio, China), 25 mM; N,N,N’,N’-Tetramethyl-p-phenylenediamine (TMPD, Cat. No. 100–22-1, Aladdin, China), 200 μM; Vitamin C (Vc, Cat. No. 50–81-7, Yuanye Bio, China), 2 mM) were then introduced, and the mixture was vortexed vigorously prior to a 1-minute incubation. Vancomycin and thioridazine (TZ) were used as the negative and positive controls, respectively, with three replicate wells per condition. The reaction was initiated by adding ADP to a final concentration of 1 mM and potassium phosphate buffer to 10 mM, and the mixture was incubated at 37°C for 20 minutes. Thereafter, 100 μL of the reaction mixture was transferred to a 96-well plate, mixed with an equal volume of BacTiter-Glo working reagent, and incubated for 20 minutes in the dark before luminescence measurement. Fluorescence intensity was measured using a multimode microplate reader (PerkinElmer, USA). ATP levels in intact bacterial cells were determined as previously described [16].

### CETSA

Cells were cultured to an OD_600_ of 0.6 and harvested by centrifugation. The resulting pellets were resuspended in lysis buffer (50 mM Tris-HCl, 500 mM NaCl, 5% glycerol, pH 8.0) and disrupted by sonication. After sonication, the lysate was centrifuged at 12,000 rpm for 10 minutes at 4°C to remove cell debris, and the supernatant was collected as the whole-cell lysate. The whole-cell lysate was first divided into two batches. One batch was supplemented with 300 μM NADH, 20 μM FAD, and 30 μM MD and then incubated at 4°C for 30 minutes, whereas the other batch served as a substrate-free control. Each batch was subsequently divided into two equal aliquots, one of which was incubated with CT and the other with an equal volume of vehicle. After incubation at 4°C for 30 minutes, the lysates were distributed into 50 μL aliquots and heated at the indicated temperatures for 3 minutes in a thermostatic metal bath (Jingxin, China), followed by cooling at 4°C for 3 minutes. Samples were then centrifuged at 8000 rpm for 10 minutes at 4°C to remove precipitated proteins. The supernatants were transferred to new microtubes and analyzed by sodium dodecyl sulfate polyacrylamide gel electrophoresis (SDS-PAGE) followed by western blot analysis.

### SDS-PAGE and western blot

Proteins were separated by SDS-PAGE in Tris-glycine running buffer and subsequently transferred onto PVDF membranes (Cat. No. IPVH00010, Merck Millipore, Germany) using a rapid transfer system (Bio-Rad, USA). Membranes were blocked with blocking buffer (Cat. No. AR0041, Boster, USA) and incubated with a mouse monoclonal anti-His tag antibody (Cat. No. HA600030, HUABIO, China), followed by a goat anti-mouse IgG secondary antibody (Cat. No. BA1050, Boster, USA). All antibodies were diluted in antibody dilution buffer (Cat. No. AR1017, Boster, USA). Protein transfer, membrane blocking, antibody incubation, and washing procedures were performed according to the manufacturers’ instructions. Immunoreactive bands were visualized using the Omni-ECL^TM^ Universal Chemiluminescence Detection Kit (Cat. No. SQ203L, Epizyme Biotech, China). Chemiluminescent signals were captured using a ChemiScope imaging system (CLiNX, China), and band intensities were quantified using ImageJ software.

### Gentamicin-selected small colony mutants and whole genome sequencing

Overnight cultures of *S. aureus* ATCC 43,300 were inoculated at 1% into LB medium containing 1/2 × MIC gentamicin and grown at 37°C with shaking until the logarithmic phase. Cultures were adjusted to an OD_600_ of 0.1, serially diluted, and plated onto LB agar supplemented with 4 × MIC gentamicin, followed by incubation for 20 hours. SCVs were selected and purified by repeated subculturing under the same selective conditions. To evaluate phenotype stability in the absence of antibiotic selection, individual SCV isolates were subsequently passaged three times on antibiotic-free LB agar plates. Colony morphology, including colony size, pigmentation, and hemolytic activity, was assessed after each passage. Among the isolated SCVs, MK-auxotrophic strains were obtained under anaerobic conditions. Growth curves of *S. aureus* ATCC 43,300 and its derived mutants were determined as described in reference [19]. Whole-genome sequencing was performed as described in reference [20] to identify the mutation sites. The auxotrophic complementation assay was performed as previously described [21].

### Membrane potential (ΔΨ) and inner membrane integrity measurement

Bis-(1,3-dibutylbarbituric acid) trimethine oxonol (DiBAC_4_(3), 10 mM, Cat. No. HY-101892, MCE, USA) is an anionic potential-response membrane probe that has been previously used to monitor changes in membrane polarization [22]. An overnight culture was inoculated (1%, v/v) into fresh LB medium and grown to mid-log phase. Cells were diluted to an OD_600_ of 0.2 and harvested by centrifugation at 5,000 × g for 5 minutes. The pellets were washed and resuspended in sterile PBS (0.01 M, Cat. No. BL601A, Biosharp, China) containing 10 mM glucose. DiBAC_4_(3) was added to a final concentration of 10 μM, and samples were incubated at 37°C in the dark for 30 minutes. PI (1 mg/mL, Cat. No. BL708A, Biosharp, China) was added to a final concentration of 30 μM, and samples were incubated at 37°C in the dark for 20 minutes. Stained bacteria were assayed in a BD Accuri C6 flow cytometer (Becton, Dickinson and Company, USA) with a laser emitting at 485 nm or 492 nm. Fluorescence intensity was measured using a multimode microplate reader (PerkinElmer, USA) at an excitation wavelength of 485/492 nm and an emission wavelength of 520/635 nm. Because CT caused substantial interference with DiBAC_4_(3) fluorescence, DiOC_2_(3) (3 mM, Cat. No. B34950, Thermo, USA) was used to assess the effects of CT on the ΔΨ of the indicated strains, according to a previously described method [16].

### Statistical analyses

All experiments were performed in biological triplicates (*n* = 3) unless otherwise specified, and data were presented as means ± SD. Statistical analysis was carried out using Graphpad Prism 10 (Graph Pad Software, USA). T test was used to calculate *p* values and significant differences (*, *p* < 0.05; **, *p* < 0.01; ***, *p* < 0.001; and ****, *p* < 0.0001). Two-way ANOVA was performed for multi-group comparisons, followed by Šidák’s post hoc multiple comparisons test.

## Results

### Overexpression of ndhC in *S. aureus* enhances sensitivity to CT

Mutation or altered expression of target genes represents a crucial adaptive strategy employed by bacteria to withstand antimicrobial pressure. In *M. tuberculosis*, for instance, mutations within the promoter region of *ndhA* can lead to overexpression of NDH-2 and thereby reduce susceptibility to NDH-2 inhibitors [4,23]. Point mutations in the coding sequence of *ndh* may also lead to drug resistance by diminishing binding between the enzyme and its inhibitors [24]. As the sole enzyme linking NADH oxidation to membrane quinone reduction in the respiratory chain, NDH-2 also carries out essential functions in the energy metabolism of *S. aureus*. The genome of *S. aureus* encodes two non-proton-pumping NDH-2, designated NdhC and NdhF [25]. Of these two isoforms, NdhC acts as the major contributor to NADH dehydrogenase activity and plays a dominant role in supporting cellular growth and regulating virulence. NdhF, by contrast, likely provides compensatory physiological functions under specific environmental stress conditions [9].

**Figure 1.**
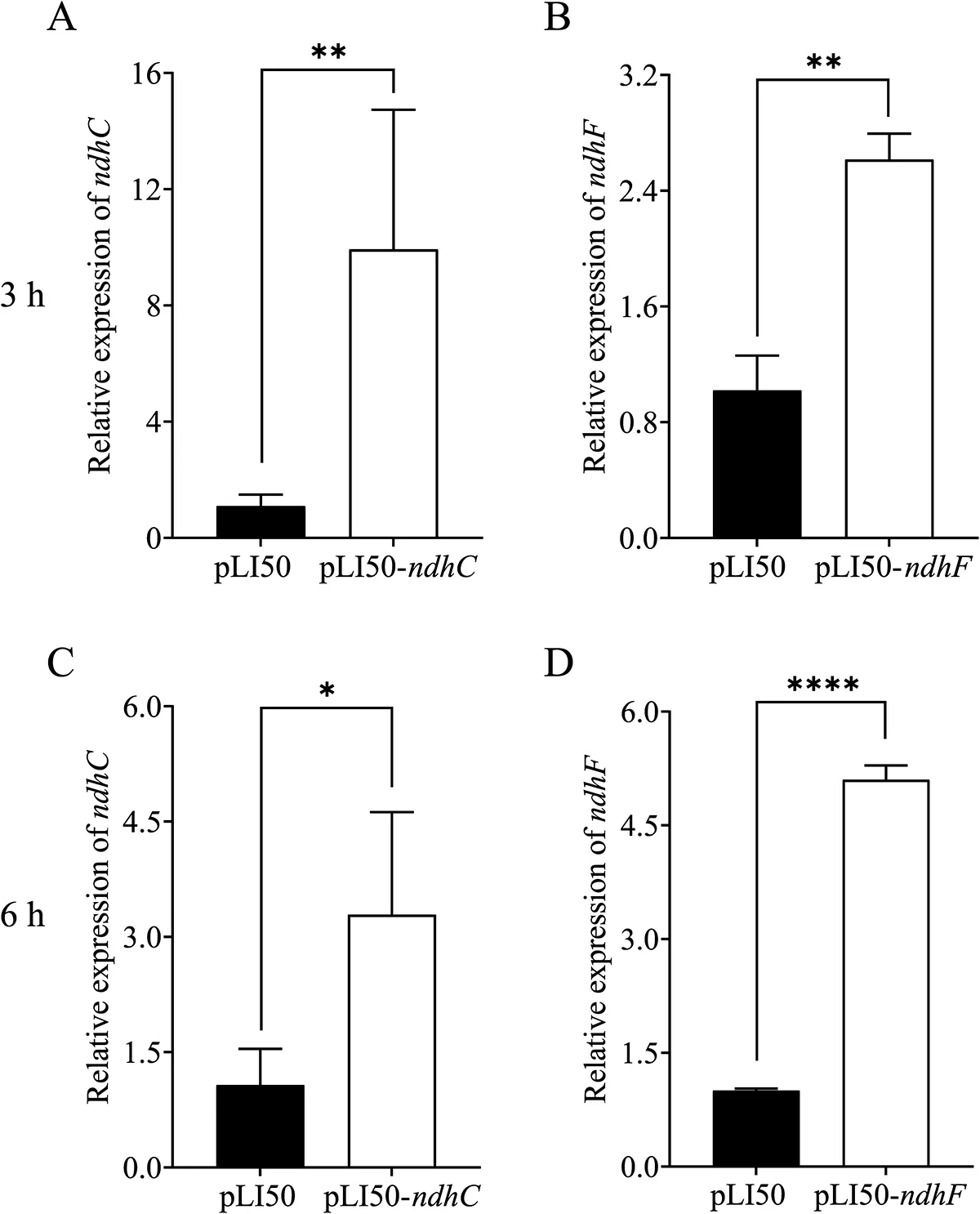
Expression levels of *ndhC* and *ndhF* in *S. aureus*. Changes in the transcript levels of *ndhC* (A, C) and *ndhF* (B, D) in *S. aureus* overexpressing *ndhC* or *ndhF* were analyzed relative to the pLI50 control at 3 h (A, B) and 6 h (C, D). Relative expression levels were quantified by RT-qPCR.

To investigate whether CT exerts its antibacterial activity in *S. aureus* by targeting NDH-2, we previously attempted to isolate CT-resistant mutants but were unsuccessful. We therefore constructed *ndhC*- and *ndhF*-overexpression strains directly using the pLI50 plasmid, and assessed the growth-inhibitory activity of CT against these genetically modified strains. The RT-qPCR analysis, presented in Figure 1, revealed that the mRNA levels of *ndhC* were increased approximately 9‑fold (Figure 1(A)) and 3‑fold (Figure 1(C)) at 3 and 6 hours of incubation, respectively, in the *ndhC*-overexpressing strain compared with the control strain harboring the empty plasmid. Similarly, the transcript levels of *ndhF* were elevated around 3‑fold (Figure 1(B)) and 5‑fold (Figure 1(D)) at the same time points in the *ndhF*-overexpressing strain. These results indicated successful and significant upregulation of the target genes in both the *ndhC*- and *ndhF*-overexpressing strains.

To assess how overexpression of NDH-2 influences the antibacterial activity of CT, we determined the MICs of CT against *S. aureus* strains overexpressing either NdhC or NdhF via broth microdilution. As summarized in Table 1, overexpression of *ndhC* conferred a two-fold reduction in the MIC for CT and its structural analog dihydrotanshinone (DHT). In contrast, overexpression of *ndhF* had no effect on the susceptibility to either compound compared to the vector control. Parallel susceptibility testing was performed against two established NDH-2 inhibitors, 2-heptyl-4-hydroxyquinoline N-oxide (HQNO) and TZ. These compounds displayed comparable patterns of altered susceptibility in the *ndh*‑*2*‑overexpressing strains, mirroring the response to CT. *ndhC* overexpression also increased susceptibility to antibiotics with distinct targets, including tetracycline, penicillin, and rifampicin, while *ndhF* overexpression again had no effect (Table 1). In contrast, susceptibility to vancomycin remained unchanged in both the *ndhC*- and *ndhF*-overexpressing strains compared to the control. Collectively, these data suggest that *ndhC* overexpression induces a general metabolic perturbation rather than a specific response to NDH-2 inhibitors. This phenomenon prompted us to further investigate the target of CT using orthogonal biochemical approaches, as described below.

**Table 1.** MICs of CT against *ndhC*- and *ndhF*-overexpressing *S. aureus* RN4220 strains.

Compound	MIC (μg/mL)		
	pLI50	pLI50-*ndhC*	pLI50-*ndhF*
Cryptotanshinone	4	2	4
Dihydrotanshinone	16	8	16
HQNO	8	4	8
Thioridazine	16	8	16
Tetracycline	8	4	8
Penicillin	0.0625	0.03125	0.0625
Rifampicin	0.125	0.0625	0.125
Vancomycin	1	1	1

### CT selectively inhibits NdhC over NdhF

**Figure 2.**
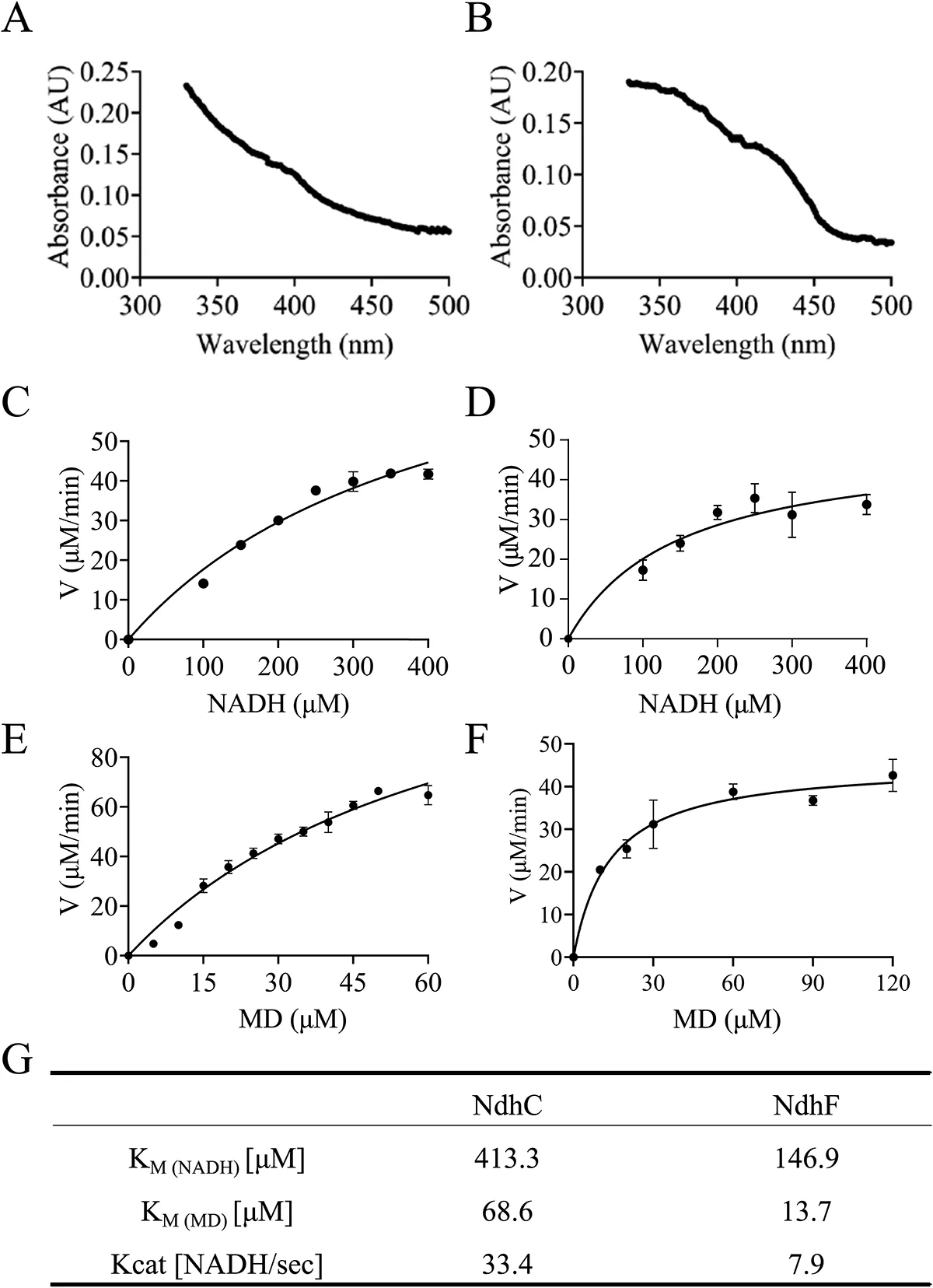
Spectral and enzymatic properties of heterologously expressed NDH-2. (A, B) UV-visible spectra of NdhC (A) and NdhF (B). (C, D) Michaelis‑Menten kinetics of NdhC (C) and NdhF (D) with NADH as the substrate. (E, F) Michaelis‑Menten kinetics of NdhC (E) and NdhF (F) with MD as the substrate. (G) kinetic parameters (Km and Kcat) were determined.

Given the distinct functional roles of NdhC and NdhF in basal metabolism and pathogenic adaptation, both proteins were subjected to heterologous expression and purification. Recombinant NdhC and NdhF were successfully expressed and purified following previously established protocols in our laboratory [18]. To evaluate protein integrity and cofactor occupancy, UV‑visible spectroscopy was performed. No characteristic absorption peak of FAD^+^ was detected at 450 nm in either protein preparation (Figure 2(A,B)), indicating loss of non-covalently bound FAD during purification. Exogenous FAD was therefore included in all subsequent enzymatic assays.

Enzyme kinetic measurements were carried out in abuffer containing 500 mM NaCl and 5% glycerol, using varying concentrations of NADH and water-soluble quinone MD as substrates. For recombinant NdhC, the Michaelis constant (Km) for NADH was 413.3 μM with a maximal velocity (Vmax) of 90.84 μM/min (Figure 2(C)), and the Km for MD was 68.6 μM with a Vmax of 149.0 μM/min (Figure 2(E)). The corresponding catalytic constant (Kcat) for NADH oxidation was calculated as 33.4 s^−1^ (Figure 2(G)). In comparison, NdhF exhibited a Km of 146.9 μM and Vmax of 49.6 μM/min for NADH (Figure 2(D)), and a Km of 13.7 μM and Vmax of 45.63 μM/min for MD (Figure 2(F)), with a Kcat of 7.9 s^−1^ for NADH turnover (Figure 2(G)). The catalytic constant for NADH oxidation was markedly higher for NdhC than for NdhF. These results are consistent with previous reports [25,26], and support the use of purified enzymes in subsequent inhibition studies.

**Figure 3.**
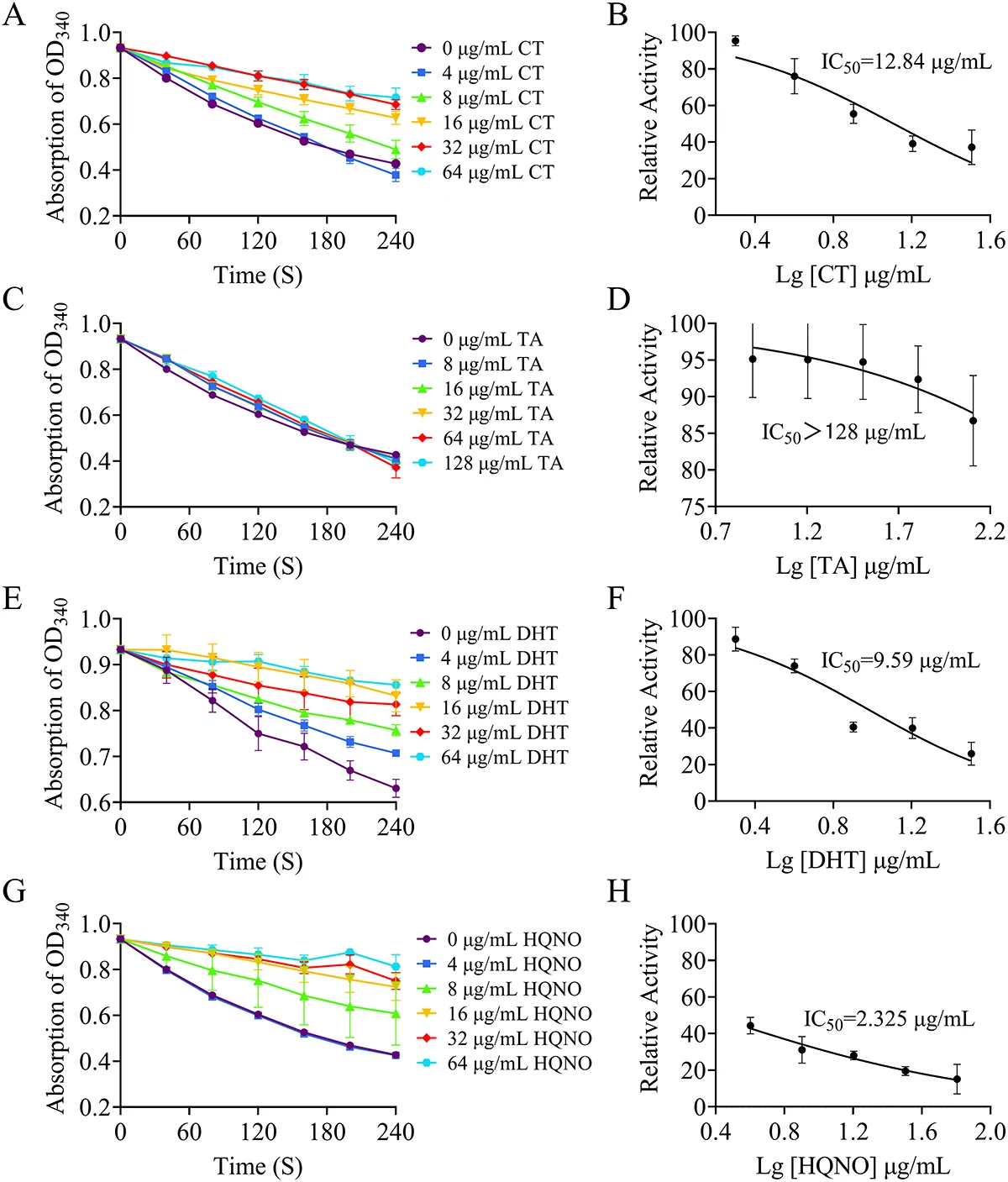
The effect of CT on enzyme activity of NdhC. Concentration of NADH was fixed at 300 µM for all experiments. A variable slope model was fitted to determine the IC_50_ values. In each panel, NADH:quinone oxidoreductase activities in the absence of drugs were used for 100%. (A) the NADH UV absorbance at 340 nm was monitored after the addition of CT. (B) CT inhibition curves for NdhC in the presence of 30 µM MD. (C) the NADH UV absorbance at 340 nm was monitored after the addition of TA. (D) TA inhibition curves for NdhC in the presence of 30 µM MD. (E) the NADH UV absorbance at 340 nm was monitored after the addition of DHT. (F) DHT inhibition curves for NdhC in the presence of 30 µM MD. (G) the NADH UV absorbance at 340 nm was monitored after the addition of HQNO. (H) HQNO inhibition curves for NdhC in the presence of 30 µM MD.

**Figure 4.**
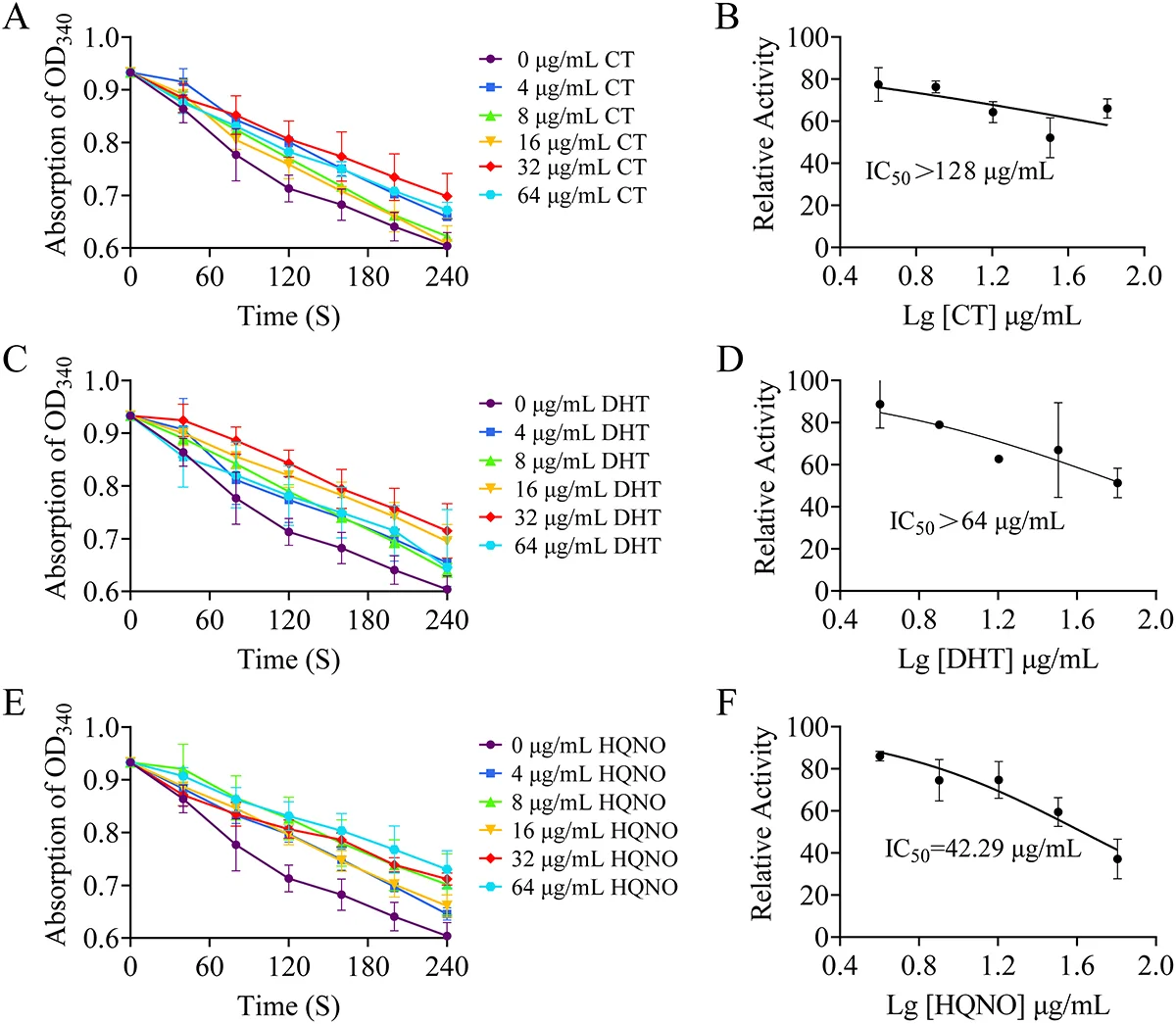
The effect of CT on enzyme activity of NdhF. Concentration of NADH was fixed at 300 µM for all experiments. A variable slope model was fitted to determine the IC_50_ values. In each panel, NADH:quinone oxidoreductase activities in the absence of drugs were used for 100%. (A) the NADH UV absorbance at 340 nm was monitored after the addition of CT. (B) CT inhibition curves for NdhF in the presence of 30 µM MD. (C) The NADH UV absorbance at 340 nm was monitored after the addition of DHT. (D) DHT inhibition curves for NdhF in the presence of 30 µM MD. (E) The NADH UV absorbance at 340 nm was monitored after the addition of HQNO. (F) HQNO inhibition curves for NdhF in the presence of 30 µM MD.

We next evaluated the *in vitro* inhibitory activity of CT against NdhC and NdhF, with IC_50_ determined from dose-response curves. HQNO was included as a positive control. CT displayed potent inhibitory activity against NdhC (Figure 3(A)), with IC_50_ values (Figure 3(B)) similar to those of HQNO (Figure 3(G,H)). By contrast, CT showed little to no inhibition of NdhF (Figure 4(A,B)), while HQNO exhibited moderate inhibition of this subunit (Figure 4(E,F)). We also examined the inhibitory effects of two structural analogs of CT, tanshinone IIA (TA) and DHT. DHT displayed a similar inhibitory profile to CT, with strong activity against NdhC (Figure 3(E,F)) but not NdhF (Figure 4(C,D)), whereas TA showed no significant inhibition of either isoform (Figure 3(C,D), Table 2). The *in vitro* inhibitory potency of CT and its analogs against NdhC showed a correlation with their whole‑cell antibacterial activity against *S. aureus* (Table 2). Compounds showing stronger NdhC inhibition also imposed more potent growth inhibition on the bacterium. This correlation supports the involvement of NdhC as a target of CT.

**Table 2.** IC_50_ values of cryptotanshinone and its analogs against NdhC and NdhF, and MICs against *S. aureus* strains.

Drug	Structure	NdhC IC_50_		NdhF IC_50_		MIC (μg/mL)		
						ATCC 43,300	USA300	Newman
		μM	μg/mL	μM	μg/mL			
Cryptotanshinone	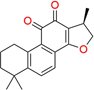	43.5	12.8	>216	>128	2	4	4
Tanshinone IIA	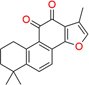	>435	>128	>435	>128	>128	>128	>128
Dihydrotanshinone	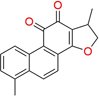	32.4	9.6	>216	>64	2	1	2
HQNO	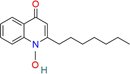	8.9	2.3	163	42.3	16	16	8

### CT acts as an uncompetitive inhibitor of NdhC with respect to MD

**Figure 5.**
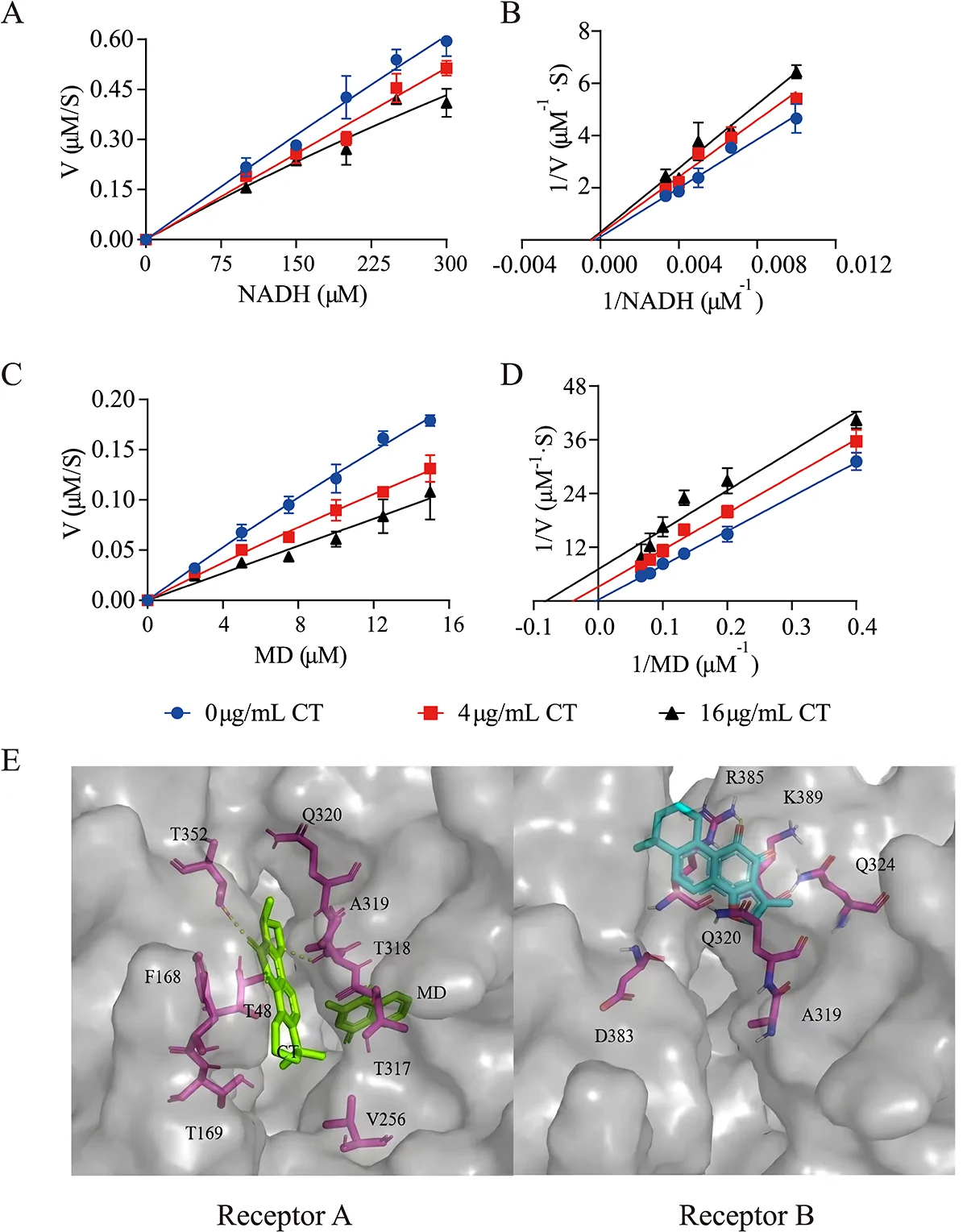
CT functions as an uncompetitive inhibitor of NDH‑2 against its substrate MD. (A, C) Michaelis‑Menten plot for the inhibition of NDH‑2 by CT. (B, D) Lineweaver‑Burk plot for the inhibition of NDH‑2 by CT. (E) Electronic cloud plot of NDH-2 and the interaction of CT with NDH-2 (PDB ID: 5NA1). Receptor A, with bound MD retained; receptor B, with MD removed.

Steady-state kinetic analyses were performed to determine the mode of inhibition. When NADH was varied as the substrate at fixed saturating MD, CT exhibited a noncompetitive inhibition pattern with respect to NADH. This was evidenced by a dose-dependent decrease in Vmax without a significant change in the apparent Km for NADH (Figure 5(A,B)). In contrast, at a fixed NADH concentration and variable MD concentrations, addition of CT led to coordinate decreases in both Vmax and the apparent Km for MD (Figure 5(C,D)). This kinetic signature is characteristic of uncompetitive inhibition, in which the inhibitor preferentially binds the enzyme-substrate complex to form a catalytically inactive ternary complex, resulting in both increased apparent substrate affinity and reduced maximal velocity. Collectively, these data suggest that CT acts as a noncompetitive inhibitor with respect to NADH and an uncompetitive inhibitor with respect to MD.

To further characterize the inhibitory mechanism of CT against *S. aureus* NdhC, molecular docking analysis was performed based on the crystal structure of NdhC (PDB ID: 5NA1). Given the uncompetitive behavior observed in kinetic assays, which suggests binding dependent on enzyme-substrate complex formation, docking was carried out using the NdhC structure containing MD as the primary receptor (receptor A). A parallel docking analysis was performed using the ligand-free NdhC structure (receptor B) as a control. As shown in Figure 5(E) and Table S3, CT localized near the active site in both receptor models, but its binding mode and stability differed markedly. In the NdhC-MD complex, CT was stably positioned in a conserved hydrophobic pocket adjacent to MD, forming extensive hydrophobic interactions with F168, T169, V256, T317, T318, and Q320, as well as hydrogen bonds with A319 and T352. The predicted binding free energy was −8.28 kcal/mol, suggesting favorable binding affinity. In the MD-free model, by contrast, the binding pose of CT was shifted. Hydrophobic contacts were formed primarily with A319, Q320, Q324, D383, and K389, with only one hydrogen bond to R385, and the binding free energy increased to −7.81 kcal/mol. These docking results are consistent with our enzyme kinetic data and support the uncompetitive inhibition mode for CT with respect to the quinone substrate of NdhC.

### T352 mediates high‑affinity binding of CT to the NdhC-MD complex

Molecular docking analysis suggested that A319 and T352 contribute critically to the stable binding of CT within the NdhC-MD complex (receptor A), as both residues participate in hydrogen-bonding interactions with CT. In contrast, R385 was identified as the sole residue forming a hydrogen bond with CT in the ligand-free enzyme model (receptor B). To investigate the functional contributions of these candidate residues to CT-mediated inhibition of NdhC and to distinguish their substrate-dependent binding roles, five NdhC point mutants (A319S, A319E, T352A, T352E, and R385A) were constructed by whole-plasmid PCR-based site-directed mutagenesis. After sequence verification, the resulting plasmids were transformed into *E. coli* BL21 (DE3) for recombinant expression. As shown in Figure S1, cells expressing the T352A, T352E, or R385A mutants displayed a characteristic yellow-green color upon IPTG induction, similar to the wild-type enzyme, indicating proper folding and FAD cofactor incorporation [18,27]. In contrast, cultures producing A319S or A319E mutants appeared creamy white, suggesting impaired FAD binding. Subsequent purification attempts yielded only very low levels of soluble target protein for these two variants, preventing the preparation of sufficient pure protein for kinetic characterization. As a result, sufficient purified protein samples for enzymatic kinetic analysis could not be obtained. Notably, A319 resides within the highly conserved quinone-binding motif ^319^AQXAXQ^324^, which is critical for maintaining the structural integrity of the substrate-binding pocket and overall conformational stability in NDH-2 enzymes [26]. Mutation at A319 may therefore disrupt local folding or cofactor coordination, leading to protein aggregation, degradation, or sequestration in insoluble fractions, and thus severely compromising soluble expression.

**Figure 6.**
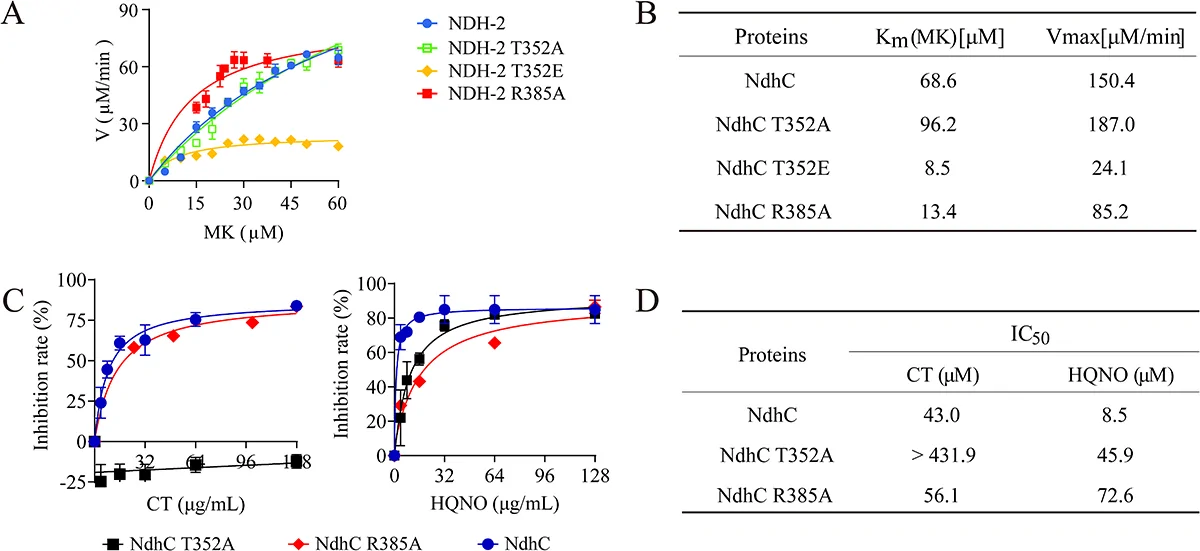
Characterization of NdhC mutants. (A) Michaelis‑Menten plots of reaction rate (V) vs substrate concentration. (B) Kinetic parameters (Km and Vmax) of the enzymes were determined. (C) CT inhibition of NdhC mutants. (D) IC_50_ values of CT and HQNO for wild-type NdhC and its mutants, measured in the presence of 30 µM MD.

To obtain reliable inhibition kinetics, we characterized the NADH dehydrogenase activity of the soluble NdhC mutants T352A, T352E, and R385A. As shown in Figure 6(A,B), both the T352A and R385A variants exhibited catalytic activity similar to that of the wild-type enzyme, indicating that these mutations did not compromise the enzyme’s structural or functional integrity. In contrast, the T352E mutant showed markedly reduced activity, suggesting that this residue is critically sensitive to charge-mediated conformational changes. Accordingly, T352A and R385A were selected for downstream inhibition assays: T352A eliminates the hydrogen-bond donor capacity of Thr352 without compromising enzyme activity, ideal for examining its role in CT binding, while R385A serves as a functional control to assess the contribution of this residue. As shown in Figure 6(C,D), the R385A mutant exhibited similar CT sensitivity to wild-type NdhC (IC_50_ = 56.1 μM), suggesting on inhibitory potency. Conversely, the T352A mutation drastically elevated the IC_50_ of CT from 43.0 μM (wild-type) to >431.9 μM, indicating the critical role of residue T352 in mediating CT’s inhibitory activity. The control HQNO inhibited wild-type and both mutant enzymes equivalently, consistent with mechanisms between HQNO and CT. Collectively, these docking and kinetic data suggest T352 as a key residue for specific, favorable CT binding within the NdhC-MD complex. These findings further support the uncompetitive inhibition mechanism of CT toward NdhC.

### CT acts at NdhC in isolated membrane preparations in a substrate-dependent manner

Although the *in vitro* enzymatic assays demonstrated that CT selectively inhibits NdhC, whether CT engages NdhC as its direct target in a cellular context remained to be established. To address this, we performed a series of orthogonal assays.

**Figure 7.**
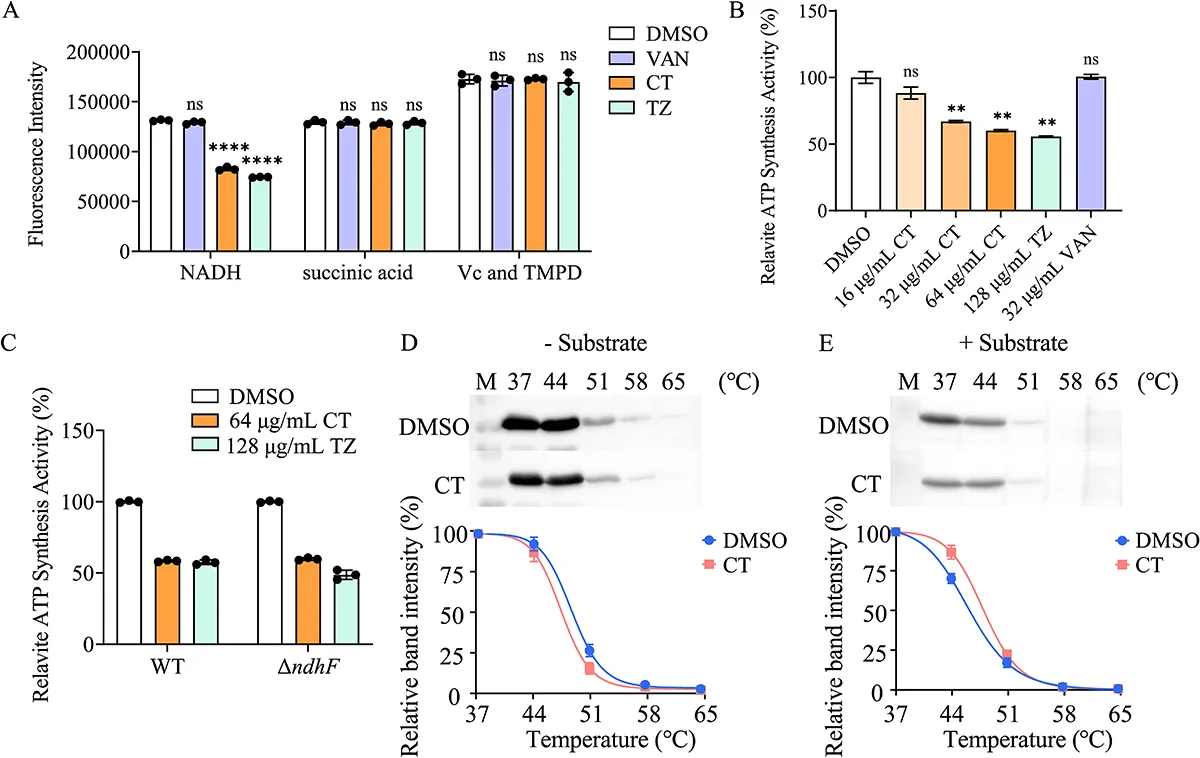
CT directly engages NdhC in the bacterial membrane. (A) ATP synthesis was measured in inverted membrane vesicles isolated from *S. aureus* ATCC 43,300. Vesicles were energized with NADH, succinate, or ascorbate/TMPD. CT was added at 64 μg/mL. Thioridazine (TZ, 128 μg/mL) and vancomycin (VAN, 32 μg/mL) served as positive and negative controls, respectively. (B) membrane vesicles were energized with NADH and treated with increasing concentrations of CT. (C) membrane vesicles were prepared from wild-type (ATCC 43,300) and Newman Δ*ndhF* strains and energized with NADH. (D) CETSA was performed using lysates of *E. coli* BL21 (DE3) expressing His-tagged NdhC. Lysates were incubated with DMSO (1.6%) or CT (8 μg/mL). (E) CETSA was performed as in (D), except that lysates were incubated with NADH and MD prior to treatment with DMSO (1.6%) or CT (8 μg/mL). NdhC present in the supernatant was quantified by western blotting, and band intensities at each temperature were normalized to the 37°C value. Statistical analysis was performed using two-way ANOVA with Šidák’s post hoc test. ****p < 0.0001 (44°C). Data are presented as mean ± SD from three independent experiments (*n* = 3), except for CETSA, which was conducted in two independent experiments (*n* = 2, ± SEM).

Inverted membrane vesicles prepared from *S. aureus* were used to assess CT’s inhibitory effect on ATP synthesis driven by different electron donors. CT dose-dependently inhibited NADH-driven ATP synthesis but had no effect on succinic acid or Vc/TMPD-driven ATP synthesis (Figure 7(A,B)). This inhibition pattern mirrored that of TZ, a known NDH-2 inhibitor, suggesting that CT acts on the NDH-2-mediated electron transport pathway in its native membrane environment. To further examine whether this inhibitory effect is specifically mediated by NdhC rather than the other isoform NdhF, an *ndhF* knockout *S. aureus* strain was generated. Inverted membrane vesicles prepared from this *ndhF* mutant exhibited similar sensitivity to CT as wild-type vesicles, with comparable inhibition of NADH-driven ATP synthesis (Figure 7(C)), ruling out the involvement of NdhF and supporting the involvement of NdhC in the observed inhibition.

Furthermore, a CETSA was performed using *E. coli* lysates expressing His-tagged NdhC to provide direct biophysical evidence for the binding between CT and NdhC. CT significantly increased the thermal stability of NdhC when NADH and MD (the electron donor and the quinone substrate, respectively) were present (Figure 7(D,E)). No thermal stabilization was observed in the absence of substrates. This substrate-dependent stabilization supports that CT engages NdhC in a manner requiring formation of the enzyme-substrate complex, consistent with an uncompetitive inhibition mechanism.

Together, these orthogonal data suggest that CT acts at the NdhC-dependent segment of the electron transport chain (ETC) in isolated membrane preparations, and that this interaction is substrate-dependent.

### Scvs with respiratory deficiency exhibit collateral sensitivity to CT

**Figure 8.**
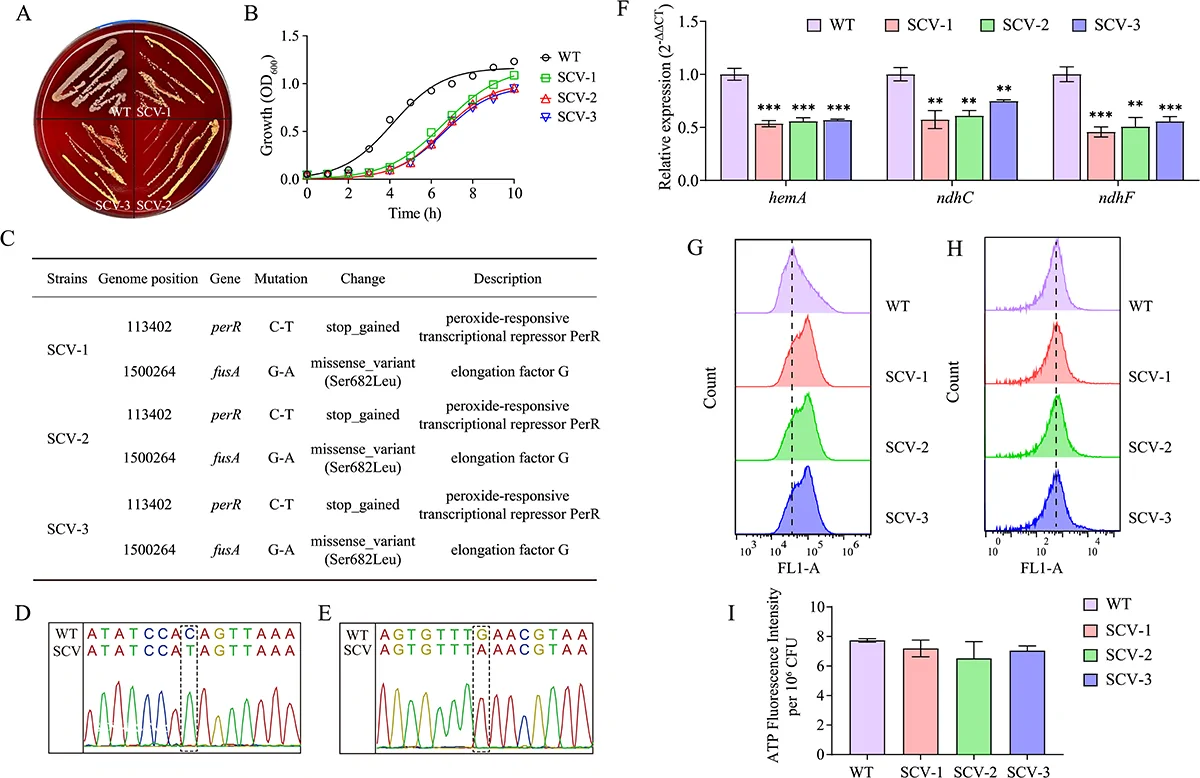
Phenotypic characterization of gentamicin-induced *S. aureus* ATCC 43,300 SCVs isolated under aerobic conditions. (A) growth characteristics of the parent strain and its SCVs. (B) growth curves of the parent strain and SCVs. (C) single nucleotide polymorphisms (SNPs) identified in mutant strains compared to the parent strain. (D, E) sequencing analysis of the mutation sites, with black dashed boxes highlighting the locations of the mutations. (F) relative expression of *hemA, ndhC*, and *ndhF* in the parent strain and SCVs. (G, H) assessment of membrane potential (G) and inner membrane integrity (H) in the parent strain and SCVs. (I) intracellular ATP levels of the parent strain and SCVs. ATP fluorescence intensity was normalized to 10^6^ CFU.

*S. aureus* SCVs are frequently induced by aminoglycoside therapy and are closely associated with persistent and recurrent infections [28]. To investigate the susceptibility changes of SCVs to CT, a stable SCV model was established via induction with subinhibitory concentrations of gentamicin, and three genetically stable SCV isolates were obtained following multiple rounds of mutagenesis and screening. Relative to the parental strain, these SCV isolates displayed notably smaller colony diameters (approximately one-tenth of the wild-type), retarded growth kinetics, enhanced staphyloxanthin production, and significantly reduced hemolytic activity (Figure 8(A,B)). Antimicrobial susceptibility testing results (Table 3) revealed that the MIC values of conventional antibiotics including gentamicin, cephalexin, tetracycline, and fusidic acid against SCVs were at least 4-fold higher than those against the parental strain. In stark contrast, the MIC values of CT, HQNO, and TZ against SCVs were reduced by 2-fold or more. Although the magnitude of MIC reduction (≥2-fold) is smaller than the ≥4-fold increase observed for conventional antibiotics, the consistent decrease for all three NDH-2 inhibitors suggests a distinct, heightened susceptibility of SCVs to this inhibitor class.

**Table 3.** MICs of cryptotanshinone and other antimicrobial agents against *S. aureus* ATCC 43,300 SCV strains.

Drugs	MIC (μg/mL)						
	WT	SCV-1	SCV-2	SCV-3	SCV-4	SCV-5	SCV-6
Gentamicin	0.5	2	2	2	4	4	4
Cephalexin	2	8	8	16	1	1	1
Tetracycline	0.5	2	2	2	0.5	0.5	0.5
Fusidic acid	2	>64	>64	>64	<0.25	<0.25	<0.25
Vancomycin	1	1	1	1	1	1	1
Cryptotanshinon	2	1	1	1	1	1	1
Thioridazine	32	16	16	16	16	16	8
HQNO	8	2	2	2	4	4	4

To elucidate the molecular basis of SCV formation, whole-genome sequencing analysis was performed on the three SCV isolates and their parental strain. Comparative genomic analysis (Figure 8(C)) identified two identical single-nucleotide mutations shared by all three SCV strains. The first was a C-to-T nonsense mutation at position 113,402 of the *perR* gene (encoding a peroxide-responsive transcriptional repressor), resulting in premature translation termination. The second was a G-to-A missense mutation (Ser682Leu) at position 1,500,264 of the *fusA* gene, which encodes elongation factor G (EF-G). Subsequent confirmation of each variation was achieved through PCR amplification and sequencing (Figure 8(D,E)). Notably, residue Ser682 lies within the ribosome-binding domain V (aa 606–693), and missense mutations at this site are known to be associated with the SCV phenotype in *S. aureus* [21]. According to the model proposed by Norström et al., such mutations impair EF-G-ribosome interaction, which leads to altered regulation of downstream signaling pathways (e.g. the ppGpp pathway) and, consequently, disruption of heme biosynthesis. This in turn triggers ETC dysfunction and a reduction in ΔΨ. Consistent with this model, we observed that in the *fusA perR* SCVs, the expression levels of *hemA*, *ndhC*, and *ndhF* were all significantly downregulated compared to the parental strain (Figure 8(F)). Moreover, these SCVs exhibited a marked reduction in ΔΨ without any detectable compromise in membrane integrity (Figure 8(G,H)) [29], together with a trend toward decreased ATP synthesis (Figure 8(I)). Of note, however, none of the SCVs displayed auxotrophy for hemin, MD, or thymidine (Figure S2).

**Figure 9.**
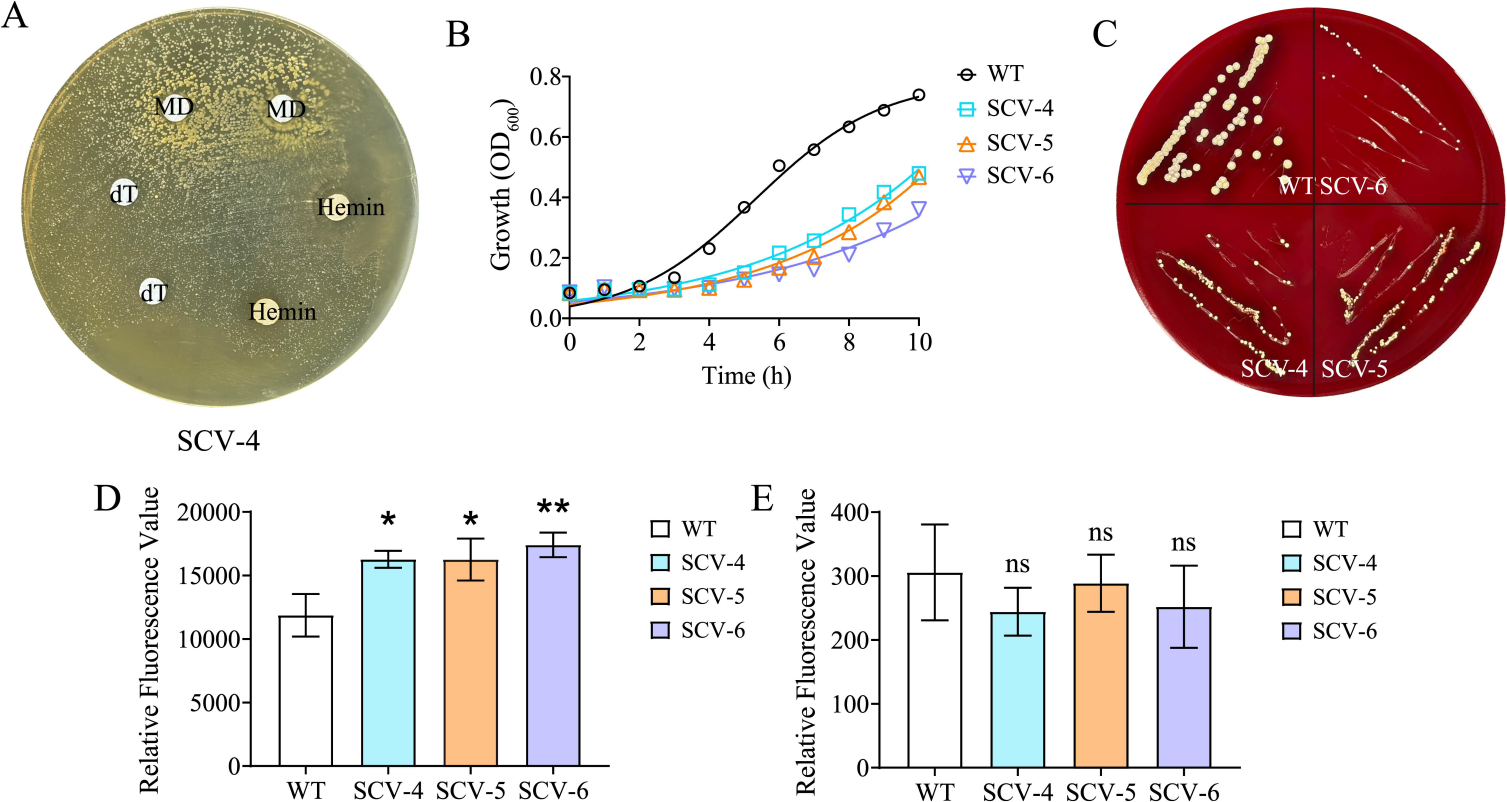
Phenotypic characterization of gentamicin-induced *S. aureus* ATCC 43,300 SCVs isolated under anaerobic conditions. (A) auxotrophy test of *S. aureus* 43,300 SCVs on agar plates. A representative result for SCV-4 is shown. Plates were supplemented with menadione (MD), hemin, or thymidine (dT) as indicated. (B) growth curves of the parent strain and SCVs. (C) growth characteristics of the parent strain and its SCVs. (D, E) assessment of membrane potential (D) and inner membrane integrity (E) in the parent strain and SCVs.

To generate an additional SCV background, we isolated MK auxotrophs by selecting for gentamicin-resistant colonies under anaerobic conditions. Three independent MD-dependent SCVs were obtained (Figure 9(A)). These isolates displayed the hallmarks of MK-deficient SCVs, including slow growth (Figure 9(B)), reduced hemolytic activity (Figure 9(C)), and significantly reduced ΔΨ without compromising membrane integrity (Figure 9(D,E)). As expected, these MK auxotrophs showed an 8-fold increase in gentamicin resistance but exhibited increased susceptibility to CT, HQNO, and TZ compared to the parental strain (Table 3). Notably, in contrast to the *fusA perR* SCVs, the MK auxotrophs also displayed increased susceptibility to fusidic acid and cephalexin, while their susceptibility to tetracycline and vancomycin remained unchanged.

**Figure 10.**
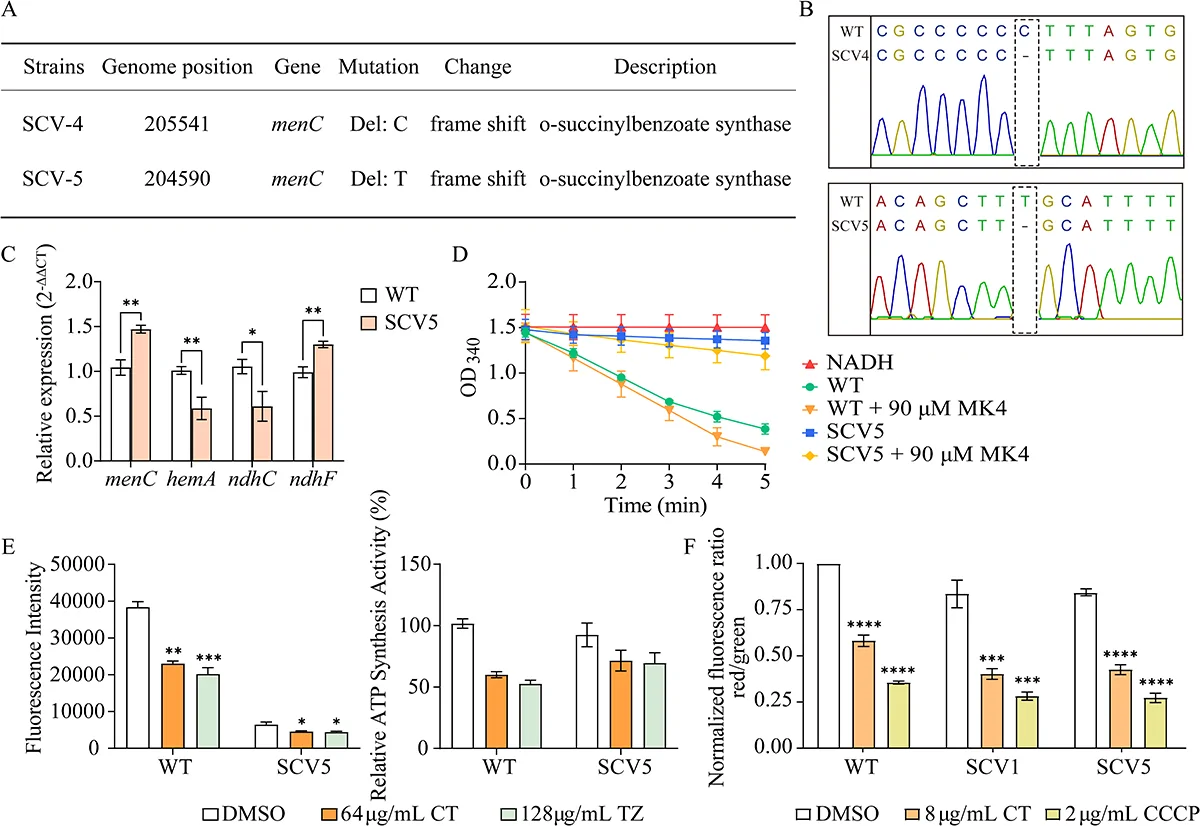
Genetic and functional characterization of the menaquinone auxotrophic SCVs. (A) identification of frameshift mutations in the *menC* gene of SCV-4 and SCV-5 by whole-genome sequencing. (B) sequencing chromatograms confirming the deletion mutations in SCV-4 and SCV-5 compared with the parental strain. Black dashed boxes indicate the mutation sites. (C) relative transcript levels of *menC, hemA, ndhC*, and *ndhF* in the parental strain and SCV-5, determined by RT-qPCR. (D) NADH dehydrogenase activity in inverted membrane vesicles from the parental strain and SCV-5, measured in the presence or absence of exogenous menaquinone-4 (MK4, 90 μM). Activity was monitored by the decrease in absorbance at 340 nm. NADH concentration was fixed at 400 μM. (E) effects of CT on NADH-driven ATP synthesis in inverted membrane vesicles from the parental strain and SCV-5. (F) effects of CT on membrane potential in the parental strain and SCV-5, assessed using the fluorescent dye DiOC_2_(3).

To further characterize the MK auxotrophic mutants and assess whether NDH-2 retains residual activity despite MK deficiency, we performed whole-genome sequencing on the three independently isolated SCVs. In SCV-4 and SCV-5, we identified deletion mutations at genomic positions 205,541 (C) and 204,590 (T), respectively, both resulting in frameshift mutations in the *menC* gene, as confirmed by PCR verification (Figure 10(A,B)). Notably, SCV-4 exhibited phenotypic instability with frequent reversion to the wild-type phenotype; therefore, subsequent characterization focused on the stable SCV-5 isolate. SCV-6, by contrast, exhibited no detectable genomic mutations, most likely due to reversion to the wild-type that had occurred prior to sequencing, and was excluded from further analysis.

RT-qPCR analysis revealed that in SCV-5, transcript levels of *ndhC* and *hemA* were significantly downregulated compared to the parental strain, yet remained readily detectable, while *menC* and *ndhF* transcripts were upregulated (Figure 10(C)). To directly assess whether NDH-2 retains enzymatic function in this MK-deficient background, we measured NADH dehydrogenase activity using inverted membrane vesicles prepared from SCV-5. While the residual NADH dehydrogenase activity was markedly reduced compared to the parental strain, a low but measurable activity was consistently detected (Figure 10(D)). Moreover, the addition of exogenous MK4 enhanced this residual activity in vesicles from both SCV-5 and the wild-type strain, confirming the functional dependence of the remaining NDH-2 pool on quinone cofactor availability (Figure 10(D)). Consistent with the retention of residual NADH dehydrogenase activity, SCV-5 inverted membrane vesicles supported NADH-driven ATP synthesis, albeit at a reduced level relative to the wild-type. Importantly, this ATP synthesis was partially inhibited by CT, indicating that the residual NDH-2 activity in MK auxotrophs remains functionally accessible to CT (Figure 10(E)). Furthermore, CT treatment similarly dissipated the ΔΨ in SCV-5 cells as it did in the parental strain (Figure 10(F)), corroborating the functional engagement of CT with the residual ETC in these variants.

To determine whether the increased susceptibility of MK auxotrophs to CT is specifically dependent on the NdhC-MD complex, we performed a complementation assay by supplementing the growth medium with exogenous MD. As shown in Table S4, addition of MD fully restored the CT MIC in SCV-5 to a level comparable to that of the parental strain. This observation is consistent with the notion that the CT-sensitizing effect in MK auxotrophs is at least partially dependent on the formation of the NdhC-MD complex.

## Discussion

Targeting the bacterial respiratory chain represents a promising strategy to counteract the metabolic adaptation and antibiotic tolerance associated with *S. aureus* SCVs. A critical gap, however, exists in understanding the *in vivo* relevance and druggability of distinct NDH-2 isoforms in this pathogen. Here, we show that CT is a potent inhibitor of *S. aureus* NDH-2 and delineate its unique mechanism of action. Biochemical and mutational analyses reveal that CT acts preferentially on the NdhC isoform, with its activity dependent on prior MK binding and critically involving the Thr352 residue. To further explore whether CT engages NdhC in a more physiologically relevant context, we performed a series of orthogonal assays. Membrane vesicle experiments demonstrated that CT specifically inhibits NADH-driven ATP synthesis, and this inhibition was unaffected by *ndhF* knockout, supporting the involvement of NdhC. Furthermore, CETSA showed that CT stabilized NdhC in the presence of NADH and MD, while no stabilization was detected without these substrates, consistent with substrate-dependent binding. This inhibition mechanism appears to exploit a key metabolic vulnerability. Specifically, *fusA perR* SCVs isolated in this study, characterized by downregulation of *hemA*, *ndhC*, and *ndhF* and a concomitant reduction in ΔΨ without loss of membrane integrity, exhibited marked collateral sensitivity to NDH-2 inhibitors. While these SCVs displayed increased tolerance to several conventional antibiotics, their susceptibility to CT, HQNO, and TZ was significantly enhanced. This observation was further validated using an independent SCV background, MK auxotrophs, which also showed increased susceptibility to NDH-2 inhibitors, albeit with a broader hypersensitivity profile. The co-occurrence of a diminished ΔΨ and heightened sensitivity to NdhC inhibition suggests a potential functional linkage between respiratory chain activity, membrane energetics, and the efficacy of this inhibitory mechanism. We acknowledge that the *in vitro* enzyme assays used MD rather than the physiological MK substrate, and that purified NdhC lacks the native membrane environment, which may influence inhibitor sensitivity. Nevertheless, our membrane vesicle experiments offer additional support in a more physiological context.

Why do SCVs with reduced ΔΨ exhibit heightened susceptibility to CT and other NDH-2 inhibitors? We propose an energy threshold hypothesis, in which a preexisting energetic deficiency lowers the cellular tolerance for additional ETC inhibition. In wild-type *S. aureus*, the ETC possesses sufficient reserve capacity to withstand partial inhibition of NDH-2. However, in SCVs, the downregulation of multiple ETC-related genes compromises respiratory capacity and reduces ΔΨ (Figure 8(G)). Under this stressed condition, further inhibition of residual NDH-2 activity by CT may push the cells below a viable energy threshold, resulting in heightened susceptibility. This model is consistent with our observation that MK auxotrophs, which have a more severe respiratory defect, also show increased susceptibility to CT and other NDH-2 inhibitors. Moreover, consistent with this hypothesis, we found that *fusA perR* SCVs exhibited a trend toward reduced ATP synthesis, suggesting an overall decline in energy production. This model is further corroborated by a recent study in *S. aureus* demonstrating that energy metabolism-defective mutants and clinical SCVs display increased susceptibility to lugdunin, a compound that targets bacterial membrane energetics [13]. Similar observations have also been reported in *M. tuberculosis*, where NDH-2 inhibitors show enhanced activity against non-replicating and metabolically compromised bacteria [4,30].

While MK auxotrophy severely compromises overall NADH dehydrogenase activity, our data suggest that a residual functional NdhC pool may persist in SCV-5 under the standard culture conditions employed in this study, as evidenced by the observation that NADH-driven ATP synthesis in SCV-5 membrane vesicles, although markedly reduced, remained partially susceptible to CT inhibition. Given that CT selectively inhibits NdhC but not NdhF in our biochemical assays, this partial inhibition points to the presence of functionally active NdhC in these MK-deficient variants. The retention of residual NdhC activity is possibly attributable to trace amounts of MK/ubiquinone precursors or analogs present in rich media such as LB, TAB, or MH, which might support low but detectable NdhC function despite the *menC* frameshift mutation. This residual activity, although marginal, appears sufficient to support CT binding and inhibition, which may help reconcile the observed collateral sensitivity of MK auxotrophs with the proposed NdhC-targeting mechanism. The concurrent upregulation of *ndhF* in SCV-5 could represent a compensatory response to respiratory stress; however, given the isoform specificity of CT for NdhC over NdhF, this upregulation is unlikely to account for the CT sensitivity phenotype. Taken together, these findings are consistent with the energy threshold model, in which even partial inhibition of the residual NdhC pool in energetically compromised SCVs may be sufficient to disrupt the already marginal ETC flux and trigger collateral sensitivity. Nevertheless, we acknowledge that direct evidence for NdhC as the primary intracellular target of CT remains to be fully established, and alternative or additional targets cannot be formally excluded.

The formation of *S. aureus* SCVs is highly heterogeneous and can arise from transient phenotypic adaptation or stable, mutation-driven genetic alterations [31]. Among stably inherited SCVs, approximately 20% of clinical and laboratory isolates carry mutations in four major metabolic pathways. Defects in the MK biosynthetic pathway (*menD*, *menB*, *menC*) or heme biosynthetic pathway (*hemB*, *hemH*, *hrtA*) impair electron transport and represent the most common SCV types recovered from chronic infections such as osteomyelitis and prosthetic joint infections [32]. Thymidylate synthase (*thyA*)-deficient SCVs are frequently associated with cystic fibrosis [33], and mutations in fatty acid biosynthesis (*acpP*, *fabF*, *accD*) are often identified in clinical isolates from patients undergoing long-term antimicrobial therapy [34]. Dysregulation of global regulators including *agr*, *sigB*, *mgrA*, and *codY* also contributes to the stability of the SCV phenotype [35]. Recent clinical studies have further linked specific genotypes to distinct infection contexts: *mpsB* mutations give rise to CO_2_-dependent SCVs in chronic infections [36], while *sufB* mutations drive SCV development and persistent infection in recurrent prosthetic joint infections [37]. The SCV strains characterized in this study harbored both a nonsense mutation in *perR* and a missense mutation in *fusA*. Although *perR* mutations have not previously been associated with *S. aureus* SCVs, they have been shown to directly induce small colony phenotypes in *Listeria* [38]. Notably, *fusA* mutations have been detected in *S. aureus* SCVs isolated from patients with atopic eczema [39], which may lend relevance to the SCV model used in this study.

While NDH-2 is a promising anti-infective target, most studies have focused on *M. tuberculosis*, with limited inhibitors identified for *S. aureus* [4,40]. Here, we show that CT is a potent inhibitor of *S. aureus* NdhC, acting preferentially on this isoform with an uncompetitive mechanism with respect to MD in a substrate-dependent manner, and that its activity critically involves the T352 residue. This finding provides insight into the molecular recognition of CT by NdhC and offers structural cues that may inform the rational design of future NDH-2-targeted therapeutics. Mechanistically, we propose an energy threshold hypothesis in which the preexisting respiratory deficiency of SCVs lowers their tolerance for additional NDH-2 inhibition, rendering them collaterally sensitive to this class of compounds. This model finds support in the behavior of our *fusA perR* SCVs and MK auxotrophs, which display heightened susceptibility to CT and other NDH-2 inhibitors under the tested conditions. Although not every SCV type has been experimentally confirmed to exhibit reduced ΔΨ, the majority of clinically relevant SCVs, particularly those with defects in MK or heme biosynthesis, are characterized by impaired electron transport. Based on the energy threshold hypothesis proposed above, such SCVs would be predicted to display heightened susceptibility to NDH-2 inhibitors, including CT. We acknowledge, however, that our current evidence for NdhC as the primary intracellular target of CT is derived largely from in *vitro* and isolated membrane systems; direct demonstration of target engagement in intact bacterial cells remains an open question. Whether the energy threshold hypothesis extends to a broader range of clinical SCV isolates also warrants further investigation.

## Supplementary Material

supplement_clean.docx

## Data Availability

The data that support the findings of this study are openly available in figshare at https://doi.org/10.6084/m9.figshare.31989249, reference number.
