# Supplementary material for: Cryptotanshinone inhibits Staphylococcus aureus NdhC via uncompetitive inhibition: Collateral sensitivity in respiratory-deficient SCVs
Source: Virulence. 2026 Aug 24;17(1):2723683. doi: 10.1080/21505594.2026.2723683 (PMC13532008; doi:10.1080/21505594.2026.2723683)
Supplement: Supplementary file 1 [file KVIR_A_2723683_SM0804.docx]

**Table S1.** Strains and plasmids used in this study.

| Strains or plasmids | Genotype or description | Sources |
| --- | --- | --- |
| **strain** |  |  |
| *S. aureus* ATCC 43300 | ST5, CC5, *spa* t007, SCC*mec* II, *pvl*⁻, MRSA | ATCC* |
| *S. aureus* ATCC 43300-SCV-1 | Gentamicin-induced spontaneous resistant mutant derived from ATCC 43300 | Spontaneous resistant mutant, see Methods 2.13 |
| *S. aureus* ATCC 43300-SCV-2 | Gentamicin-induced spontaneous resistant mutant derived from ATCC 43300 | Spontaneous resistant mutant, see Methods 2.13 |
| *S. aureus* ATCC 43300-SCV-3 | Gentamicin-induced spontaneous resistant mutant derived from ATCC 43300 | Spontaneous resistant mutant, see Methods 2.13 |
| *S. aureus* ATCC 43300-SCV-4 | Gentamicin-induced spontaneous resistant mutant derived from ATCC 43300 | Spontaneous resistant mutant, see Methods 2.13 |
| *S. aureus* ATCC 43300-SCV-5 | Gentamicin-induced spontaneous resistant mutant derived from ATCC 43300 | Spontaneous resistant mutant, see Methods 2.13 |
| *S. aureus* ATCC 43300-SCV-6 | Gentamicin-induced spontaneous resistant mutant derived from ATCC 43300 | Spontaneous resistant mutant, see Methods 2.13 |
| *S. aureus* ATCC RN4220 | ST8, CC8, *spa* t211, *mec*⁻, *rsbU*⁻, *agr*⁻, MSSA | ATCC* |
| *S. aureus* ATCC RN4220-pLI50 | ATCC RN4220 containing plasmid pLI50; Cm^R^ | see Methods 2.3 |
| *S. aureus* ATCC RN4220-pLI50-*ndhC* | ATCC RN4220 containing plasmid pLI50-*ndhC*; Cm^R^ | *ndhC* overexpression strain, see Methods 2.3 |
| *S. aureus* ATCC RN4220-pLI50-*ndhF* | ATCC RN4220 containing plasmid pLI50-*ndhF*; Cm^R^ | *ndhF* overexpression strain, see Methods 2.3 |
| *S. aureus* ATCC 25923 | Wild type strain | ATCC* |
| *S. aureus* USA300 | ST8, CC8, *spa* t008, SCC*mec* IVa, *agr* I, *pvl*⁺, MRSA | ATCC* |
| *S. aureus* Newman | Wild type strain | ATCC* |
| Δ*ndhF* | *S. aureus* Newman Δ*ndhF* | Knockout via CRISPR-Cas system, see Methods 2.4 |
| *E. coli* DH5α | F– φ80lacZΔ M15 Δ (*lacZYA-argF*) *U169 recA1 endA1 hsdR17* (rK^–^, mK^+^) *phoA supE44* λ- *thi–1 gyrA96 relA1* | Stratagene** |
| *E. coli* DH5α-pLI50 | DH5α containing plasmid pLI50; Ap^R^ | see Methods 2.3 |
| *E. coli* DH5α-pLI50-*ndhC* | DH5α containing plasmid pLI50-*ndhC*; Ap^R^ | *ndhC* overexpression strain, see Methods 2.3 |
| *E. coli* DH5α-pLI50-*ndhF* | DH5α containing plasmid pLI50-*ndhF*; Ap^R^ | *ndhF* overexpression strain, see Methods 2.3 |
| *E. coli* BL21 (DE3) | F– *omp*T *hsdS_B_* (rB^–^, mB^–^) *gal dcm* (DE3) | Sangon Biotech† |
| *E. coli* BL21 (DE3)-pET28a (+)-*ndhC* | BL21 (DE3) containing plasmid pET28a (+)-*ndhC*; Km^R^ | [1] |
| *E. coli* BL21 (DE3)-pET28a (+)-*ndhF* | BL21 (DE3) containing plasmid pET28a (+)-*ndhF*; Km^R^ | *ndhF* heterologous expression strain, see Methods 2.3 |
| *E. coli* BL21 (DE3)-pET28a (+)-*ndhC-*A319S | BL21 (DE3) containing plasmid pET28a (+)-*ndhC-*A319S; Km^R^ | *ndhC* mutant strain, see Methods 2.3 |
| *E. coli* BL21 (DE3)-pET28a (+)-*ndhC-*A319E | BL21 (DE3) containing plasmid pET28a (+)-*ndhC-*A319E; Km^R^ | *ndhC* mutant strain, see Methods 2.3 |
| *E. coli* BL21 (DE3)-pET28a (+)-*ndhC-*T352A | BL21 (DE3) containing plasmid pET28a (+)-*ndhC-*T352A; Km^R^ | *ndhC* mutant strain, see Methods 2.3 |
| *E. coli* BL21 (DE3)-pET28a (+)-*ndhC-*T352E | BL21 (DE3) containing plasmid pET28a (+)-*ndhC-*T352E; Km^R^ | *ndhC* mutant strain, see Methods 2.3 |
| *E. coli* BL21 (DE3)-pET28a (+)-*ndhC-*R385A | BL21 (DE3) containing plasmid pET28a (+)-*ndhC-*R385A; Km^R^ | *ndhC* mutant strain, see Methods 2.3 |
| **Plasmids** |  |  |
| pET28a (+) | T7 expression vector; Km^R^ | Miaoling Bio †† |
| pET28a (+)-*ndhC* | pET28a (+)::*ndhC*; Km^R^ | [1] |
| pET28a (+)-*ndhF* | pET28a (+)::*ndhF*; Km^R^ | Constructed using Gibson Assembly, see Methods 2.3 |
| pET28a (+)-*ndhC-*A319S | pET28a (+)::*ndhC-*A319S; Km^R^ | Constructed using Gibson Assembly, see Methods 2.3 |
| pET28a (+)-*ndhC-*A319E | pET28a (+)::*ndhC-*A319S; Km^R^ | Constructed using Gibson Assembly, see Methods 2.3 |
| pET28a (+)-*ndhC-*T352A | pET28a (+)::*ndhC-*A319S; Km^R^ | Constructed using Gibson Assembly, see Methods 2.3 |
| pET28a (+)-*ndhC-*T352E | pET28a (+)::*ndhC-*A319S; Km^R^ | Constructed using Gibson Assembly, see Methods 2.3 |
| pET28a (+)-*ndhC-*R385A | pET28a (+)::*ndhC-*A319S; Km^R^ | Constructed using Gibson Assembly, see Methods 2.3 |
| pLI50 | pMB1; Ap^R^; Cm^R^ | Miaoling Bio †† |
| pLI50-*ndhC* | pLI50::*ndhC*; Ap^R^; Cm^R^ | Constructed using Gibson Assembly, see Methods 2.3 |
| pLI50-*ndhF* | pLI50::*ndhF*; Ap^R^; Cm^R^ | Constructed using Gibson Assembly, see Methods 2.3 |
| pnCasSA-BEC | *S.aureus* genome editing vector, Km^R^, Cm^R^. (Bacterial expression of Cas9 nuclease, tracrRNA and crRNA guide) | [2] |
| pnCasSA-BEC-sgRNA | pnCasSA-BEC-sgRNA; Cm^R^ | Knockout via CRISPR-Cas system, see Methods 2.4 |

Note: Km^R^, kanamycin resistant; Ap^R^, ampicillin resistant; Cm^R^, chloramphenicol resistance.

*****, ATCC, American Type Culture Collection, Manassas, USA.

**, Stratagene, Agilent Technologies, Santa Clara, USA.

†, Sangon Biotech, Shanghai, China.

††, Miaoling Bio, Wuhan Miaoling Biotechnology Co., Ltd., Wuhan, China.

**Table S2.** Primers used in this study.

| Primer name | Sequence (5'→3') | Explanation |
| --- | --- | --- |
| *ndhC*-F | CTTGCAGACGAATGTCGGCATCACAATCTGACTGAATCTTGCTTG | Amplification of the *ndhC* gene for overexpression |
| *ndhC*-R | CGGCTTAATAGCTCACGCTATGTACAACAATAAAGCCCTTCAGTG |  |
| *ndhF*-F | AGACGAATGTCGGCAatcccagttatgtaatagtgcctt | Amplification of the *ndhF* gene for overexpression |
| *ndhF*-R | CTTAATAGCTCACGCTAagcaatgtcagtaatagtgtgaac |  |
| pLI50-F | TAGCGTGAGCTATTAAGCCG | Amplification of linear pLI50 for overexpression |
| pLI50-R | TGCCGACATTCGTCTG |  |
| *ndhC*-YYBD-F | TATACCATGCATCATCATCATCACCATATGGCACAGGATCGCAAAAAAGTTCTGGTG | Amplification of the *ndhC* gene for heterologous expression |
| *ndhC*-YYBD-R | TAGCAGCCGGATCTCAGTGGTGGTGGTGGTGGTGCTCGAGTTAGAATTTACCTTTTTTA |  |
| *ndhF*-YYBD-F | TATACCATGCATCATCATCATCACCATATGAAAAACTTAGTTTT | Amplification of the *ndhF* gene for heterologous expression |
| *ndhF*-YYBD-R | TTAACCATTATGATATCTCGAGTTAGAATTTACCTTTTTTA |  |
| Pet-XXH-F | TGATGATGATGCATGGTATATCTCCTTCTTAAA | Amplification of linear pET28a (+) for heterologous expression |
| Pet-XXH-R | CCACCACCACCACTGAGATCCGGCTGCTAACAAAGCCCG |  |
| Pet-YZ-F | GGGCTTTGTTAGCAGCCGGATCT | Verification of *E. coli* BL21 (DE3) colonies carrying pET28a (+)-*ndhC/ndhF* |
| Pet-YZ-R | TTAACTTTAAGAAGGAGATATA |  |
| *ndhC* A319S-F | GGCGAAGAACGTCCGCTGCCGACAACCTCACAGATTGCCATGCAGCAGGGCGAAAGC | Amplification of A319S mutation fragment |
| *ndhC* A319S-R | GCTTTCGCCCTGCTGCATGGCAATCTGTGAGGTTGTCGGCAGCGGACGTTCTTCGCC |  |
| *ndhC* A319E-F | GGCGAAGAACGTCCGCTGCCGACAACCGAACAGATTGCCATGCAGCAGGGCGAAAGC | Amplification of A319E mutation fragment |
| *ndhC* A319E-R | GCTTTCGCCCTGCTGCATGGCAATCTGTTCGGTTGTCGGCAGCGGACGTTCTTCGCC |  |
| *ndhC* T352A-F | GAAGAATTTGAATATGTGGATCGCGGCGCCGTGTGTAGCCTGGGCAGCCATGATGGT | Amplification of T352A mutation fragment |
| *ndhC* T352A-R | ACCATCATGGCTGCCCAGGCTACACACGGCGCCGCGATCCACATATTCAAATTCTTC |  |
| *ndhC* T352E-F | GAAGAATTTGAATATGTGGATCGCGGCGAAGTGTGTAGCCTGGGCAGCCATGATGGT | Amplification of T352E mutation fragment |
| *ndhC* T352E-R | ACCATCATGGCTGCCCAGGCTACACACTTCGCCGCGATCCACATATTCAAATTCTTC |  |
| *ndhC* R385A-F | gcctttatgaaaaaagttatcgataccGCCgcagtttttaaaattggcggtattggt | Amplification of R385A mutation fragment |
| *ndhC* R385A-R | accaataccgccaattttaaaaactgcGGCggtatcgataacttttttcataaaggc |  |
| *ndhC*-YZ-F | GTGTGAAACGCGGCCGTATTG | Verification of site-directed mutagenesis |
| *ndhC*-YZ-R | agcttcctttcgggc |  |
| SgRNA-F-F | GAAAGATGCTCAAATTGTCTCAGT | Construction of *ndhF*-targeting sgRNA |
| SgRNA-F-R | AAACACTGAGACAATTTGAGCATC |  |
| pnCasSA-R | CCGCCCATCCGACGCTATTTG | Verification of sgRNA insertion into pnCasSA-BEC plasmid (used with SgRNA-F-F) |
| QCYZ-NdhF-F | CTTAAAATTCGCGGCCCACG | Verification of *ndhF* deletion mutants |
| QCYZ-NdhF-R | GCGGCGGATATGGTAATATG |  |
| *gyrb*-RT For | CAAATGATCACAGCATTTGGTACAG | Amplification of *gyrb* gene |
| *gyrb*-RT Rev | CGGCATCAGTCATAATGACGAT |  |
| *ndhC*-RT For | GTGAAATTAAATGGCTCAAGATCGT | Amplification of *ndhC* gene |
| *ndhC*-RT Rev | GCTTCTTCTGTTGATATCGCTT |  |
| *ndhF*-RT For | AAGATGTTCGCAT | Amplification of *ndhF* gene |
| *ndhF*-RT Rev | TCATAATCAATTTTAGAATT |  |
| *hemA-*RT For | TAGCGGCTGCAACAATTTCG | Amplification of *hemA* gene |
| *hemA-*RT Rev | TGTCCATCGTTTCCGCTTGA |  |
| *menC-*RT For | TAGCATATGGAGCGACAGCG | Amplification of *menC* gene |
| *menC-*RT Rev | GGTCGGCTTTGTTGCTTTCA |  |

**Table S3.** Binding energies of MK to NdhC.

| CT binding to NdhC without MK | | CT binding to NdhC pre-bound with MK | |
| --- | --- | --- | --- |
| Hydrogen-bonding residue | Binding free energy (kcal/moL) | Hydrogen-bonding residue | Binding free energy (kcal/moL) |
| Arg 385 | -7.81 | Ala 319；Thr 352 | -8.28 |
| Arg 385 | -7.72 | Ala 319；Lys 379 | -8.02 |
| His 51 | -7.6 | Ala 319；Lys 379 | -7.97 |
| His 51 | -7.59 | Arg 385 | -7.93 |
| Arg 385 | -7.33 | No hydrogen bonds | -7.92 |
| Gln 320 | -7.16 | Arg 385 | -7.84 |
| Thr 48；ARG 385 | -7.16 | Arg 385 | -7.83 |
| Arg 385 | -7.11 | No hydrogen bonds | -7.45 |
| Lys 379 | -7.1 | No hydrogen bonds | -7.42 |
| No hydrogen bonds | -6.71 | Ala 319 | -6.84 |

**Table S4**. MICs of CT against WT and SCV5 after MD supplementation.

| Drugs | MD^a^ | MIC (μg/mL) | |
| --- | --- | --- | --- |
|  |  | WT | SCV5 |
| Cryptotanshinone | － | 2 | 1 |
|  | ＋ | 2 | 2 |
| Thioridazine | － | 32 | 16 |
|  | ＋ | 32 | 32 |

a: 1 μg/mL.

**Figure S1.** Visual appearance of bacterial pellets expressing NdhC mutants.

**Figure S2.** Auxotrophy test of *fusA perR* SCVs on agar plates. A representative result for SCV-1 is shown. Plates were supplemented with menadione (MD), hemin, or thymidine (dT) as indicated.

**References**

[1] Zhou JL, Chen HH, Xu J, et al. Myricetin acts as an inhibitor of type II NADH dehydrogenase from *Staphylococcus aureus*. Molecules. 2024;29(10):2354. doi: 10.3390/molecules29102354.
[2] Gu T, Zhao S, Pi Y, et al. Highly efficient base editing in *Staphylococcus aureus* using an engineered CRISPR RNA-guided cytidine deaminase. Chem Sci. 2018;9(12):3248-3253. doi: 10.1039/c8sc00637g.
